# Natural Products as a Source for Treating Neglected Parasitic Diseases

**DOI:** 10.3390/ijms14023395

**Published:** 2013-02-06

**Authors:** Dieudonné Ndjonka, Ludmila Nakamura Rapado, Ariel M. Silber, Eva Liebau, Carsten Wrenger

**Affiliations:** 1 Department of Biological Sciences, Faculty of Science, University of Ngaoundere, B. P. 454, Cameroon; E-Mail: dede_ndjonka@yahoo.com; 2 Unit for Drug Discovery, Department of Parasitology, Institute of Biomedical Science, University of São Paulo, Av. Prof. Lineu Prestes 1374, 05508-000 São Paulo-SP, Brazil; E-Mails: ludmilanr@usp.br (L.N.R.); asilber@usp.br (A.M.S.); 3 Institute for Zoophysiology, Schlossplatz 8, D-48143 Münster, Germany

**Keywords:** neglected infectious diseases, natural compounds, schistosomiasis, river blindness, trypanosomatids

## Abstract

Infectious diseases caused by parasites are a major threat for the entire mankind, especially in the tropics. More than 1 billion people world-wide are directly exposed to tropical parasites such as the causative agents of trypanosomiasis, leishmaniasis, schistosomiasis, lymphatic filariasis and onchocerciasis, which represent a major health problem, particularly in impecunious areas. Unlike most antibiotics, there is no “general” antiparasitic drug available. Here, the selection of antiparasitic drugs varies between different organisms. Some of the currently available drugs are chemically *de novo* synthesized, however, the majority of drugs are derived from natural sources such as plants which have subsequently been chemically modified to warrant higher potency against these human pathogens. In this review article we will provide an overview of the current status of plant derived pharmaceuticals and their chemical modifications to target parasite-specific peculiarities in order to interfere with their proliferation in the human host.

## 1. Introduction

Infectious diseases have a significant impact in human heath stock. A variety of these diseases are caused by parasites that belong to the diseases of poverty or the so-called neglected tropical diseases. Amongst others, these include the causative agents of trypanosomiasis, leishmaniasis, schistosomiasis, lymphatic filariasis and onchocerciasis. These parasites are responsible for a high rate of mortality and morbidity each year in the respective endemic countries. Since vaccines and safe and affordable treatments are still lacking, there is an urgent need to discover novel therapeutics against these human health threats. The current situation is aggravated by the fact that most people living in the endemic countries have a low-income profile, making the pharmaceutical market for the development of new medications financially unattractive to the private-research sector. Therefore, natural sources, such as plants, represent a major opportunity to discover new lead molecules [1–4]. In this review we will shed light on the discovery and application of natural plant derived products on human neglected diseases.

## 2. Nematodes

Nematodes are roundworms that belong to the phylum Nematoda or Nemathelminthes [5]. Even though the total number of nematode species has been estimated to be over 1 million [6], only about 28,000 species have been described [7], with over 16,000 of these species being parasites of plants and animals (including humans). Parasitic nematodes constitute an enormous medical and veterinary burden in some parts of the world; in the tropics, however, they represent a severe public health problem. The most common and persistent parasitic nematodes of humans are the soil-transmitted nematodes—roundworms (*Ascaris lumbricoides*), whipworms (*Trichuris trichiura*), hookworms (*Necator americanus* and *Ancylostoma duodenale*) and thread worms (*Strongyloides stercoralis)—*and the filarial nematodes that are responsible for lymphatic filariasis (LF) (*Brugia sp*, *Wuchereria bancrofti*) and onchocerciasis (*Onchocerca volvulus*).

Onchocerciasis or subcutaneous filariasis is a parasitic disease caused by *O. volvulus*. The disease affects several millions of people in the world and is transmitted from host to host by the blood-feeding “blackfly” *Simulium*. While more than 90% of all onchocerciasis cases are found in 30 African countries, the rest occur in isolated foci that exist in Yemen and six countries in central and South America World Health Organization [8]. About 37 million persons are infected with *O. volvulus*, of whom 270,000 are blind and 500,000 visually impaired [9].

LF is a mosquito-borne infection caused by the lymphatic dwelling parasites *W. bancrofti*, *B. malayi* and *B. timori*. These parasites invade and block the lymphatic system of the infected person [10]. Lymphatic vessel dysfunction and damage ultimately lead to clinical symptoms such as lymphoedema, elephantiasis and genital pathology. The WHO estimates that 1.1 billion people live in high risk areas, with 120 million people already infected with LF [11,12]. The filarial nematode *W. bancrofti* accounts for 91% of LF infections, while *B. malayi* and *B. timori* are responsible for the remaining 9% in the Southeast Asia region.

The burden of onchocerciasis and LF causes long term disability, social stigmatization and, in the case of onchocerciasis, forces the affected population to abandon the infested areas which usually have a high agricultural potential [13,14]. Thus, a high burden of onchocerciasis primarily leads to a highly unproductive population and consequently to economic loss and slowdown of country development over the years.

During the last three decades, a lot of progress has been made in the control of onchocerciasis and LF; however, the diseases still constitute a serious public health concern in the endemic countries. From 1987 to date, the control of LF and onchocerciasis has been based on two approaches: vector control using insecticides and mass drug administration of diethylcarbamazine (DEC), ivermectin and albendazole. While ivermectin is the sole drug used in community-directed treatment of onchocerciasis, DEC or ivermectin in combinantion with albendazole is used for the global control of LF [15]. The recommended treatment of filariasis patients is the administration of albendazole combined with ivermectin [16,17]. A combination of DEC and albendazole is also effective [16]. All of these treatments are microfilaricides and only show a limited macrofilaricidal activity.

The development of anthelminthic resistance is a worldwide reality [9,18–20] and also one of the greatest challenges in worm control. Early in the last decade, resistances to insecticides and ivermectin have been reported [9,18]. Additionally, a re-infestation phenomenon was observed in insecticide-treated areas. Due to these limitations, vector control was stopped. Drugs to treat onchocerciasis and LF are commonly used in combination to reduce microfilariae in the blood and skin. However, all of them have their limits. In fact, DEC or ivermectin treatment of infected individuals can cause high adverse effects [21].

Anthelmintic resistance, secondary effects and limited macrofilaricidal activities of the known antifilarial drugs have stimulated the search for alternative treatment. Here, as an alternative source of filaricidal compounds, medicinal plants have received more attention [22]. Medicinal plants have been used as therapies in traditional health care systems since prehistoric times and are still the most important health care source for the majority of the world population [23]. It is estimated that more than 60% of the world’s population rely on traditional herbal medicine to meet their primary health care needs [24].

### 2.1. Medicinal Plants in the Treatment and Control of Filariasis

#### 2.1.1. Subcutaneous Filariasis

Subcutaneous filariasis or onchocerciasis is a parasitic disease that is caused by *O. volvulus.* The cattle parasite *O. ochengi* is the closest known relative of *O. volvulus* with which it shares the same arthropod vector, *Simulium damnosum. The O. ochengi* system fills the critical niche between laboratory studies in rodent models and field evaluation of onchocerciasis control in human populations. The cattle—*O. ochengi* model equals that of human onchocerciasis, with nodules that closely resemble those formed by *O. volvulus* [25]. Thus it is feasible that medicinal plants, traditionally used by farmers against the bovine parasite, also affect the human parasite *O. volvulus*.

Nyasse *et al*. [26] demontrated that polycarpol from *Polyalthia suaveolens* (Annonaceae) and 3-*O*-acetyl aleuritolic acid from *Discoglypremna caloneura* (Euphorbiaceae) exhibited significant inhibitory activities on the vitality of adult male worms of *Onchocerca gutturosa*.

Further studies were conducted by Cho-Ngwa *et al*. [27] on *O. ochengi*. These authors reported microfilaricidal activity of the hexane extract of *Homalium africanum* (Salicaceae) leaves, the hexane extract of *Margaritaria discoidea* (Euphorbiaciaea) roots, the methylene chloride extract of *H. africanum* leaves and the methylene chloride extract of *M. discoidea* leaves. However, none of the plants used showed macrofilaricidal activity. These Salicaceae and Euphorbiaceae are commonly used in the traditional treatment of onchocerciasis in North West Cameroon.

Using the cattle parasite *O. ochengi*, Ndjonka *et al*. [28] reported that ethanolic extracts of the bark of *Anogeissus leiocarpus* (Combretaceae) and *Khaya senegalensis* (Meliaceae) as well as leaves of *K. senegalensis* and *Euphorbia hirta* (Euphorbiaciaea) display high macro- and microfilaricidal activities, while aqueous extracts of leaves from *Parquetina nigrescens* (Asclepiadaceae) and *Annona senegalensis* (Annonaceae) displayed a more moderate effect on the worms viability. This was also observed using *Caenorhabditis elegans*, a highly suitable and free-living model organism for research on nematode parasites. Furthermore, some selected plants showed toxicity not only against wildtype *C. elegans* but also against drug resistant (ivermectin, levamisole and albendazole) strains [29]. Here, the most promising plant was *A. leiocarpus* (Combretaceae) with a high toxicity against *O. ochengi*, *C. elegans* wildtype and *C. elegans* drug resistant strains [29]. The phytochemical analysis of an *A. leiocarpus* extract showed a high amount of tannins, which have been reported to have a certain anthelmintic activity [30,31]. Tannins present in their composition several phenolic groups such as ellagic, gallic and gentisic acids. Gallic and gentisic acids have been reported to be toxic for *C. elegans* [30]. Recently, we showed that ellagic acid exhibited higher toxicity against *C. elegans* wildtype and drug resistant strains (Ndjonka and Liebau, personal communication). High microfilaricidal and macrofilaricidal activities were also reported with ellagic acid [29].

Thomsen *et al*. [32] used the anthelmintic active extracts of *Hagenia abyssinica* (Rosaceae) to develop a simple and inexpensive bioassay against the non-parasitic nematode *C. elegans*.

Katiki *et al*. [33] demonstrated that extracts rich in hydrolysable tannins such as *Acer rubrum* (Aceraceae), *Rosa multiflora* (Rosaceae) and *Quercus alba* (Fagaceae), or extracts rich in both hydrolysable and condensed tannins such as *Rhus typhina* (Anacardiaceae), were significantly more lethal to adult of *C. elegans* than extracts containing only condensed tannins such as *Lespedeza cuneata* (Fabaceae), *Salix X sepulcralis* (Salicaceae) and *Robinia pseudoacacia* (Fabaceae).

Our online search on medicinal plants used against onchocerciasis found nine publications since 2002, where a total of 17 plant species, belonging to 10 different families, have been studied. In these studies, only five pure compounds were isolated (Table 1).

#### 2.1.2. Lymphatic Filariasis

LF is a parasitic disease that is caused by *Brugia* sp. and *W. bancrofti.* Current control programs outside sub-Saharan Africa use DEC plus albendazole or DEC alone, while in Africa ivermectin plus albendazole is used because of contraindications for DEC in patients infected with *O. volvulus* [16,17]. However, there is no effective drug that targets the adult stage of the worms. Based on the observation that filarial worms depend on the endosymbiontic bacteria *Wolbachia* for metabolic and reproductive activities, doxycycline therapy is suggested by Hoerauf *et al*. [49] and Taylor *et al*. [50] for individual drug administration in bancroftian filariasis.

Several plants have been assessed for their antifilarial activity against *Brugia sp* and *W. bancrofti*. Lakshmi *et al*. [34] conducted a study on the antifilarial activity of a marine red alga, *Botryocladia leptopoda* (Rhodymeniaceae) against experimental infections with the rodent filarial parasites *Litomosoides sigmodontis* and *Acanthocheilonema viteae* and the human-pathogenic *B. malayi*. They show that the ethanolic crude extract and its hexane fraction reduce microfilarial levels and kill a significant proportion of the adult worms from *L. sigmodontis* and *A. viteae*. In the case of *B. malayi*, the macrofilaricidal efficacy was much less than that observed in the rodent parasites, but significant sterilization of the surviving female parasites was caused by the hexane and chloroform fractions of the ethanolic crude extract of *B. leptopoda.*

Fujimaki *et al*. [35] screened and provided detailed informations about 11 medicinal plants used in Guatemala for *in vitro* macrofilaricidal activity against *B. pahangi*. Among the 11 medicinal plants, the ethanolic extract of leaves of *Neurolaena lobata* (Asteraceae) showed the highest inhibitory activity against the motility of adult worms. The *in vitro* assay of the extract of *N. lobata* showed potential macro- and micro-filaricidal activities.

Misra *et al*. [36] reported the antifilarial activity in the extract of stem portions of the plant *Lantana camara* (Verbenaceae). They showed that the ethanolic crude extract of this plant administered orally to the rodent model *Mastomys coucha* killed 43.05% of adult *B. malayi* and sterilized 76% of surviving female worms. After fractionation of the ethanolic extract of this Verbenaceae, Misra *et al*. [36] were able to show that the chloroform fraction contains 34.5% adulticidal activity along with sterilization of 66% of female worms. They also demonstrated that the activity of *L. camara* depends on the rodent host. Thus, using *Meriones unguiculatus* (gerbil) rodents as host for *B. malayi*, lead to an antifilarial activity up to 80%, whereas at the same dosage, all adult worms were killed using *M. coucha* as host. The authors also reported that *L. camara* is efficient against the *A. viteae*, exerting strong microfilaricidal (95.04%) and sterilization (60.66%) efficacy with mild macrofilaricidal action. Two compounds from *L. camara*, oleanonic acid and oleanolic acid, isolated from hexane and chloroform fractions also showed an *in vitro* activity against *B. malayi* adults. Toxicity studies of *L. camara* in rats showed that all rats remained active and healthy throughout the study [34].

Sahare *et al*. [37,51] analyzed the *in vitro* effect of *Butea monosperma* (Fabaceae), *Vitex negundo* (Lamiaceae), *Aegle marmelo* (Rutaceae) and *Ricinus communis* (Euphorbiaceae) on the motility of *B. malayi* microfilariae. They reported that aqueous extract of *B. monosperma* leaves, ethanolic extract of *V. negundo* root and ethanolic extract *A. marmelo* leaves exhibited a 100% inhibition of motility of microfilariae at 100 ng/mL concentration as compared to controls. At the same concentration, methanolic extract of *R. communis* leaves and aqueous extract of *B. monosperma* roots showed no significant activity on the motility of *B. malayi* microfilaria. Using thin layer chromatography, Sahare *et al*. [51] were able to reveal the presence of alkaloids, saponin and flavonoids in the roots of *V. negundo* and coumarin in the leaves of *A. marmelos*.

Gaur *et al*. [39] studied the antifilarial activity of *Caesalpinia bonducella* (Caesalpiniaceae) seed kernel by using cotton rats *Sigmodon hispidus* and *M. coucha* that harbor the filarial parasites *L. sigmodontis* and *B. malayi*, respectively. Oral treatment of the rats with ethanolic extract of *C. bonducella* significantly reduced the number of microfilariae and macrofilariae of *L. sigmodontis*, with up to 96% filaricidal activities and 100% female sterilizing efficacy. Furthermore, Gaur *et al*. [39] reported that the butanol and the aqueous fraction exerted 73.7% and 90% reduction of microfilariae number, respectively, and 82.5% mortality in adult worms with 100% female sterilization for both fractions, while two hexane fractions lead to 64% and 95% macrofilaricidal activity and 100% worm sterilization. However, in the *B. malayi/M. coucha* model, the hexane fraction that demonstrated high macrofilaricidal activity on *L. sigmodontis*, only showed a gradual microfilaraemia reduction and caused 80% sterilization of female parasites. These results suggest that *C. bonducella* seed kernel extract and fractions show microfilaricidal, macrofilaricidal and female-sterilizing efficacy against *L. sigmodontis* and microfilaricidal and female-sterilizing efficacy against *B. malayi*. This indicates the potential of the plant in providing a lead for new antifilarial drug development [39].

Mathew *et al*. [40] investigated the *in vitro* activity of a methanolic extract of fruits of *Trachyspermum ammi* (Apiaceae) against adult bovine filarial *Setaria digitata* worms. Using high-performance liquid chromatography, they isolated the active fraction containing a phenolic monoterpene. After characterization, the active molecule showed macrofilaricidal activity and sterilizing efficacy against *B. malayi* in *M. coucha*. After extraction of leaves of *Piper betle* (Piperaceae) in methanol, Singh *et al*. [41] studied the antifilarial activities of the crude extract, the n-hexane and chloroform fractions of this Piperaceae using *B. malayi*. They demonstrated that the methanolic crude extract, the hexane and chloroform fractions of *P. betle in vivo* contain remarkable immunomodulatory properties in BALB/c mice and enhance both humoral as well as cell-mediated immune responses. Furthermore, *in vivo* antifilarial activity of the crude methanol extract and its active fractions was assessed using *B. malayi*, experimentally maintained in *M. coucha*, and the antifilarial results correlated with the immunomodulation potential of the plant. The crude extract and its active fractions suppressed microfilaraemia, showed moderate adulticidal activity and female worm sterilization.

Sharma *et al*. [38] selected extracts of roots of *Vitex negundo*, leaves of *Butea monosperma*, *Aegle marmelos* and *Ricinus communis*, to explore the possible involvement of any oxidative mechanisms in the observed antifilarial effect. They observe the presence of polyphenolic compounds and an increase in oxidative parameters in *V. negundo*, *B. monosperma* and *A. marmelos* and conclude that the observed antifilarial action of these herbal extracts might be associated with oxidative stress.

Misra *et al*. [42] studied the antifilarial activity of fruits of *Xylocarpus granatum* (Meliaceae) against *B. malayi in vivo* using the jird model *M. unguiculatus* and *M. coucha*. The *in vitro* effect of crude aqueous extract from different parts of *X. granatum* on filarial parasites was reported earlier by Zaridah *et al*. [43]. In their studies, Misra *et al*. [42] demonstrated that the crude aqueous ethanolic extract was active *in vitro* and demonstrated a 52.8% and 62.7% adulticidal and embryostatic effect on *B. malayi in vivo*, transplanted in the jird model (*M. unguiculatus*) and *M. caucha*. After fractionation (ethyl acetate fraction, n-butanol fraction, water-soluble fraction and water-insoluble fraction), only the ethyl acetate fraction revealed *in vivo* activity. Eight pure molecules were isolated from the active fraction, with the two compounds gedunin (IC_50_ = 0.239 μg/mL, SI = 889.1) and photogedunin (IC_50_ = 0.213 μg/mL, SI = 1231.4) at 5 × 100 mg/kg by subcutaneous route revealed adulticidal efficacy, with 80% and 70% mortality of the transplanted *B. malayi.*

Sashidhara *et al*. [44] isolated galactolipid from *Bauhinia racemosa* (Caesalpinaeceae). They showed that the leaves of *B. racemosa* exhibit promising *in vitro* and *in vivo* antifilarial activity against *B. malayi* which can be attributed to the presence of one pure compound, namely (2*S*)-1,2-di-*O*-linolenoyl-3-*O*-*a*-galactopyranosyl-(1/6)-*O*-*b*-galactopy ranosyl glycerol.

Azeez *et al*. [45] identified 10 spices and medicinal plants, namely coriander (*Coriandrum sativum*, Apiaceae), cassia (*Cassia*, Fabaceae), turmeric (*Curcuma longa*, Zingiberaceae), allspice (*Pimenta dioica*, Myrtaceae), cinnamon (*Cinnamomum*, Lauraceae), strychnous (*Strychnos*, Loganiaceae), lemongrass (*Cymbopogon*, Poaceae), garlic (*Allium sativum*, Amaryllidaceae), litsea (*Litsea*, Lauraceae) and vanilla (*Vanilla*, Orchidaceae) that contain phytochemicals, with the potential to inhibit the detoxification enzyme glutathione S-transferase (GST) from *B. malayi*. Molecular docking of these compounds, followed by *in vitro* GST assay, demonstrated that linalool, alpha-pinene, strychnine, vanillin, piperine, isoeugenol, curcumin, beta-caryophyllene, cinnamic acid, capsaicin, citronellol and geraniol effectively inhibit the *B. malayi* GST and therefore mark them as lead compounds.

Kushwaha *et al*. [46] reported that chemotypical variations in *Withania somnifera* (Solanaceae) lead to differentially modulated immune responses in BALB/c mice. Furthermore, the published findings [48] on the different immunomodulatory and immunotherapeutic potentials for the crude oil of *Nigella sativa* (Ranunculaceae) seeds and its active ingredients led Kushwaha *et al*. [47] to hypothesize that immunostimulation prior to pathogen invasion might provide protection against filarial infection. They demonstrated that crude root extracts of chemotypes NMITLI-101 (101R), NMITLI-118 (118R) and NMITLI-128 (128R) as well as withanolide withaferin A of *W. somnifera* offer protection to the rodent host *M. coucha* against infection of filarial parasite *B. malayi*. They also showed that treatment with *W. somnifera* extracts negatively affected not only larval establishment in the host but also led to a defective embryogenesis in female worms. This study revealed the potent immunoprophylactic property of *W. somnifera*.

Our online search on medicinal plants used against LF found 16 publications since 2002, where a total of 24 plant species, belonging to 20 different families, have been studied on. In these studies 24 pure compounds were isolated (Table 1).

### 2.2. Schistosomiasis

Schistosomiasis (also known as bilharzia, bilharziosis or snail fever) is a chronic parasitic disease caused by trematode parasites of the genus *Schistosoma.* Schistosomiasis is endemic in 77 countries, with higher prevalence in the tropical and the subtropical belt of the globe [52]. Is it estimated that about 800 million people are at risk of infection due to their exposure to contaminated water and 390–600 million people are infected with these parasites [52–55]. There are two main clinical forms of schistosomiasis: the intestinal form, which is caused by five different species (*S. mansoni*, *S. japonicum*, *S. mekongi*, *S. guineensis*, *S. intercalatum*) and the urogenital form, which is caused by *S. haematobium* [56].

The infection of *Schistosoma* sp. occurs when the larvae of the parasites are liberated by the infected snail (intermediary host), get in contact with the human host and subsequently penetrate the skin. Once inside the human hosts, the pathogens differentiate into schistosomules, which migrate via the bloodstream to the liver and develop into male and female mature forms. After mating, the worms migrate again and relocate at the mesenteric intestinal veins or the venous plexus of the urinary system. The females release the eggs, which are able to pass epithel of the blood vessels and reach the intestinal lumen, the bladder or urethra lumen in order to be expelled by feces or urine. Some of these eggs also remain in these tissues. The damage of blood vessels, together with immune reactions against the retained eggs are responsible for the clinical forms of schistosomiasis.

The first approaches towards controlling the infection were initiated in the beginning of the 20th century; however, there is still no consensus about the best strategy for controlling this disease [57]. Traditionally, the main prevention and control strategies are based on the control of the transmitting snails of the genus *Biomphalaria*, improving the sanitation conditions and the treatment of patients [56,58,59]. It is generally agreed that no individual method is sufficient to block transmission. Consequently, combinatory approaches are considered for interruption of the life cycle of *Schistosoma* sp. [54,60–62].

Currently, a main strategy to control schistosomiasis is based on the periodic treatment of people living at risk areas with anti-schistosomicidal drugs in order to reduce morbidity and transmission [63]. Moreover, it was recently suggested that schistosomiasis, as well as some other neglected diseases could be controlled by large scale treatment of the whole population with safe and efficient drugs in areas endemic for more than one parasitic disease [63].

#### 2.2.1. Currently Available Drugs for the Treatment of Schistosomiasis

In former times, chemotherapy against *Schistosoma* sp. infection depended on antimonials. Due to serious side effect caused by these drugs, the use as schistosomicidal drugs was discontinued and currently the treatment mainly relies on three compounds: metrifonate, oxamniquine and praziquantel [64,65].

##### 2.2.1.1. Metrifonate

Metrifonate (*O*,*O*-dimetil-2,2,2-tricloro-1-hydroxyethylphosphonate) was derived from an organophosphorus insecticide (dimethyl(2,2,2-trichloro-1-hydroxyethyl phosphonate). Metrifonate is a reversible inhibitor of the acetylcholinesterase, the enzyme that is responsible for acetylcholine catabolism. This inhibitory activity at low concentrations causes a selective paralysis of the parasite’s muscles, making it susceptible of being carried out by the bloodstream to the liver (in case of *S. mansoni*) or to the lungs (in case of *S. haematobium*). Since high concentrations of metrifonate are toxic for humans due to the diminished level of erythrocitary cholinesterase activity, the use is restricted to 3 doses. Recent results indicate that following the reduction of the concentration of the drug, *S. mansoni* is able to go back to the mesenteric veins within the intestine and re-establish the infection. However, *S. haematobium* adults remain in the lung and are not able to move back to the bladder or urethra veins and re-establish the infection [66,67]. Since metrifonate is only effective against the urogenital disease, the drug is only recommended by the WHO to treat the infection of *S. haematobium* [68].

##### 2.2.1.2. Oxamniquine

Oxamniquine (1,2,3,4-tetrahydro-2-[isopropylamino]methyl(−)7-nitro-6-nitro-quinoline methanol) was reported to be substrate of a sulfotransferase that produces an ester which is able to react with nucleic acids and thereby interferes with replication and transcription processes. This drug also causes an increased motility and tegument damage, which primarily affects male worms. *S. mansoni* is more susceptible to the drug than other species. Under the effect of the drug, the male worm migrates into the liver, where the cellular immune response eliminates the pathogen. Deleterious changes are also observed in females. However, they are mostly due to the lack of male stimulation rather than to a direct effect of the drug. The absence of a human homologue renders oxamniquine effective with a low cyto-toxicity profile to human cells [69]. However, the emergence of resistant strains combined with high production cost and its restricted use for *S. mansoni*, makes the drug unusable for control campaigns.

##### 2.2.1.3. Praziquantel

Praziquantel (2-(cyclohexylearbonil-1,2,3,6,7,11-hexylhydro-4*H*-pyrazino[2,1-*a*]isoquinil-4-one) is the only drug presently available for the treatment of the three human pathogenic species of *Schistosoma* (*S. mansoni*, *S. haematobium* and *S. japonicum*). The mechanism of action is not yet well established. It was proposed that the drug induces membrane alterations, producing a Ca^2+^ entry in the muscle cells and a paralysis in the contracted state. The paralyzed parasites are then carried out by the bloodstream [70–72]. The drug also causes tegumental damage allowing the host-immune cells to reach the interior of the parasite and to eliminate it [73,74]. The drug is mainly active against the adult forms of the parasite, limiting its use against the early stages of the infection [74,75]. Praziquantel is a safe and low cost drug, and it has been used in the last 20 years for control strategies and patients’ treatment in countries such as Brazil, Cambodia, China, Egypt, Morocco and Saudi Arabia [76–78]. However, due to its use at large scale control programs, the emergency of resistance and diminished efficacy were reported [68,79–89].

In search for new lead compounds, the richness of chemical structures of metabolites available in nature is also being exploited. The identification of natural products (mainly derived from plants) with schistosomicidal activity is a valuable strategy for obtaining lead compounds to combat this health problem [90–92].

#### 2.2.2. Natural Products for the Control of Schistosomiasis

Vegetable oil extracts probably constitute the first anthelmintic products in traditional medicine. The variety of their application modes evidenced the ethno-pharmacological potential of these extracts as sources of active compounds. However, there are only a few studies which are focusing on the isolation, the identification, and eventually the validation of the active molecules within the plant extracts [93–98]. More recently, an initiative of research groups all over the world has been founded to set up plant extract screening programs [99–103]. A variety of compounds that reveal schistosomicidal activity have been identified in *in vitro* assays (Table 2). However, the extrapolation of *in vitro* results to the “*in vivo* world” is far away from being trivial. A good example for this is Vernodalin (Table 2), a sesquiterpene lactone with a promising *in vitro* activity but no *in vivo* activity [104]. Even after promising results are obtained in *in vivo* studies, the application of this drug in an endemic region must take other factors into consideration, such as the presence of other infectious pathogens. These could cause co-infections and thereby interfere with the respective treatment. For example, the antimalarial activity of Artemisinin and some derivatives has been exploited for more than 20 years [105]. More recently, the discovery and *in vivo* validation of schistosomicidal activity of artemisinin was established by observing a reduction of the parasitaemia in experimentally *S. japonicum* infected mice and the susceptibility of schistosomula to the drug [106–108]. Later, these results were also confirmed for *S. mansoni.* Within this study, an increased sensitivity of the females to the drug was also observed [109]. Although these results were promising, the use of artemisinin in regions endemic for malaria should be carefully assessed, since the application of this drug to control schistosomiasis could have the serious adverse side effect of increasing artemisinin drug resistance in malaria [110,111].

#### 2.2.3. Mode of Action and Molecular Targets in *Schistosoma*

Synthetic drugs and natural compounds with schistosomicidal activity in general lead to alterations in their behavior (disruption of mating of males and females, reduction in reproductive fitness), in the morphology or constitution of the protective tegument, or in the muscle activity of the parasite.

##### 2.2.3.1. Compounds that Disrupt Mating

Compounds such as curcumin, artesunate, artemeter, artemisina, vernodalin, piplartina, as well as avocado and soybean unsaponifiable oils, essential oils from *Plectranthus neochilus* and extracts from *Calyptridium umbellatum* cause the separation of males and females (Table 2). The mated state is a fundamental process of the parasite viability inside the human host and for establishing the infection. Only when the parasites are mated (the female is held in a groove within the male body, the gynecophoric canal) the sexual maturation and egg production occurs. The induction of separation of males and females reduces or even arrests the release of eggs, which is a relevant factor in the hepatic pathology and the transmission of the disease [112].

##### 2.2.3.2. Compounds Acting on the Tegument Structure or Composition

The tegument in *Schistosoma* sp. is involved in several vital functions such as nutrient absorption, secretion and by acting as a physical barrier against components of the humoral and cellular host-immune system [124,125]. It is constantly being renewed. Due to these facts and the previous observations that drugs affecting the tegument make the parasites more sensitive to the host immune response, make it a relevant target for drug discovery. Thus, drugs leading to tegument alterations such as vacuolization or observable surface modifications, like peeling, loss of spines or changes in protuberances are considered to be suitable for in *in vivo* testing (Table 2) [88,120]. However, beside visible modifications of the tegument, additional criteria should be used to evaluate the potential schistosomicidal activity of a drug or extract, since these alterations not always affect the survival of the parasite [123]. Due to the morphological and biochemical differences between male and female parasites, the efficiency of the drug can be gender specific. For example, in *in vivo* studies with *S. mansoni* it was observed that at a concentration of 400 mg/kg curcumine was 10-fold more active against male than female worms [99,113]. On the other hand, it was reported that artemeter was more active against females of *S. mansoni* [119,126]. Compounds targeting *Schistosoma sp.* in a sex specific manner are effective for treating the infected individuals, improving their clinical outcome and their ability of transmit the disease through the diminution of the released eggs to the environment.

##### 2.2.3.3. Compounds Acting on the Parasite Nervous System

The nervous system of *Schistosoma sp.*, as well as those of other helminthes, has been considered a relevant target for drug discovery. Some observable changes such as parasite motility were associated to interfere with the activity of neurotransmitters or neuromodulators like serotonin, dopamine, acetylcholine, epinephrine, glutamate and a variety of neuropeptides [65,127–130]. In this context, Hillman *et al.* [131] used a fluorescent analogue of acetylcholine and validated the parasite cholinergic receptors as a drug target for *S. mansoni*. For these *in vitro* experiments the worms’ motor activity is a main parameter; however, although drugs causing significant motor alterations are usually evaluated as hits, their effect is not always related to the drug action.

## 3. Trypanosomatids

Human pathogens of the genus *Leishmania* and *Trypanosoma* are restricted to several species causing Chagas disease (*Trypanosoma cruzi*), sleeping sickness (*T. brucei*) and leishmaniosis (a group of disease caused by parasites of the genus *Leishmania*). The natural transmission of the first two infections is limited to America and Africa, respectively, while the leishmaniosis is distributed around the world’s tropical and subtropical belt. Plants have been used in the traditional medicine to treat infections caused by these human pathogens [132–137]. Pathogenic trypanosomatids are digenetic organisms with complex life cycles involving an insect vector and a mammalian host (including humans). To make it more complex—with exception of *Trypanosoma brucei*—all other pathogenic trypanosomatids have at least one intracellular stage in the mammalian host, which plays a major role in the establishment of the chronic infection and/or in the pathogenesis. Thus, successful therapeutic compounds should be accessible to the respective intracellular compartment in order to target the parasite. As mentioned, *T. brucei* does not have an intracellular cycle in the mammalian host. This fact does not make the challenge easier: in the advanced stages of the disease, the parasites reach the central nervous system (CNS) and as a consequence the successful drug should ideally be able to pass the blood-brain barrier.

As the insect stages of trypanosomatids can be easily cultured in axenic media, most of tests for trypanocidal and leishmanicidal activities have been carried out in these systems. However, the evaluation of compounds on insect stages presents severe limitations due to the fact that the effect cannot be extrapolated to the mammalian stages. Thus, last-generation high throughput screening platforms were developed to evaluate the effect of compounds (particularly those against *T. cruzi* and *Leishmania spp*.) in mammalian infected cells. Until today, most extracts and plant derived compounds have been evaluated in axenic systems, some of them in *in vitro* or *ex vivo* infections systems and only a limited number of them have been evaluated *in vivo* (Table 3). It is worth mentioning that—with some exceptions—only the mode of action of cubebin and its derivatives has thus far been elucidated [138].

A variety of compounds belonging to the alkaloid family presents trypanocidal activity. Most of them are phenols, therpenoids of quinines. However, none of them can be considered as promising drug candidates yet, due to the limited *in vivo* data (Table 3). Interestingly, most of these active compounds against *Trypanosoma sp*. and *Leishmania spp*. are derived from plant species of the genus *Piper* (belonging to the family Piperaceae). The variety of the metabolites derived from this species is well documented. Compounds of the primary and secondary metabolism of Piperaceae have been isolated and several of them present a well-documented biological activity within several organisms including *S. mansoni* as mentioned above [92,103,139–141]. Compounds of the plant families Asteraceae and Lauraceae reveal also activity against trypanosomatids. However, the quantity of these compounds reported up to now, is reduced in comparison to those found in Piperaceae [91,142]. The fact that most of anti-trypanosomatid activities were found in members of a single family suggests that the search for these activities was somehow restricted. As plants can be conceived as “natural organic synthesis laboratories”, it is highly emphasized to test and isolate more extracts from plants against trypanosomatids in order to obtain new molecules to fight these neglected diseases.

## 4. Summary

The most prevalent families revealing anti-filarial activity were Fabaceae represented by five species, followed by four species of Euphrobiaceae and finally Apiaceae, Rosaceae, Annonaceae and Lauraceae which were indicated by two species. The majority of the active compounds isolated from these species revealed an aromatic structure. Among these compounds, gallic, gentisic and ellagic acids were active against *O. ochengi*. Oleanonic, oleanolic acids, gedunin, photogedunin, coumarin and withanolide were interferring with *B. malayi.* It would be important to test these compounds on other filariae to establish a common and effective antifilaricide especially in areas where co-infection occurs.

Among the compounds revealing anti-schistosomal activity, a mode of action has been proposed only for artemisinin and its derivatives. The interference of these drugs with the hemoglobin metabolism is a common feature between *Schistosoma* and *Plasmodium*. Both organisms degrade the host hemoglobin into their constituting amino acids, and the free hem is eliminated by the formation of hemozoin, a less reactive iron containing molecule. Free hem is known to be involved in the generation of a variety of different reactive oxygen species and thereby causing oxidative stress. It was proposed that the hem reacts with artemisinin which affects the proliferation of the parasite [117,156].

In general, little is known about the mode of action of natural compounds leading to anti-schistosomal as well as anti trypanosomal activities. More information on the molecular mechanism involved in the action of natural compounds is necessary to predict side effects and analyze the probability of the emergence of resistant strains.

Presently, the set-up of high throughput screening facilities allows a more efficient and rapid identification of interesting schistosomicidal compounds. In this sense, previous studies on the characterization and inhibition of relevant parasite proteins like acetylcholinesterase [67,157], cysteine protease [158] serine protease [159] and glutamatergic receptors in adult *S. mansoni* [160] are relevant to the development of new possible methods towards the identification and validation of new targets. For example, Abdulla *et al*. [158] described the viability of applying a medium-throughput phenotypic screening of adult worms and schistosomula *in vitro* with a library of known drugs to identify putative schistosomicidal drugs. Interestingly, this screening could also be used to investigate the schistosomidal activity of plant derived extracts, products and active compounds.

## Figures and Tables

**Table 1:** List of medicinal plants, products and active compounds known to have antifilarial properties.

Names	Family	Parts used	Solvent used for extraction	Active compounds	Activities	References
*Polyalthia suaveolens*	Annonaceae			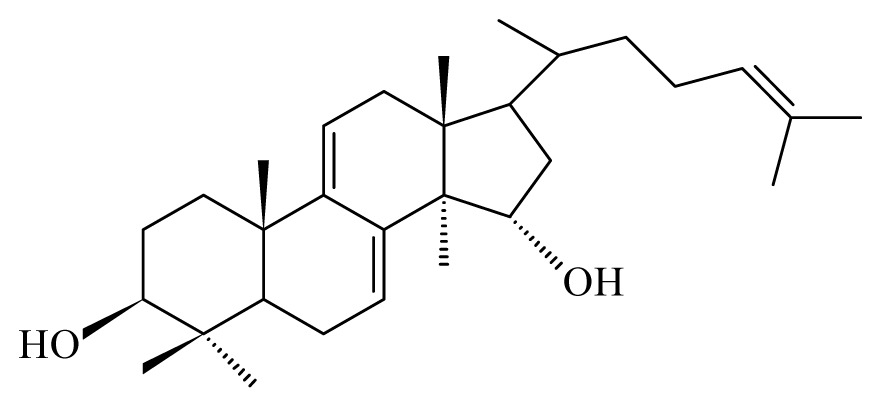 Polycarpol	*O. gutturosa*: Significant inhibitory activities on the vitality of adult male worms	[26]
*Discoglypremna caloneura*	Euphorbiaceae			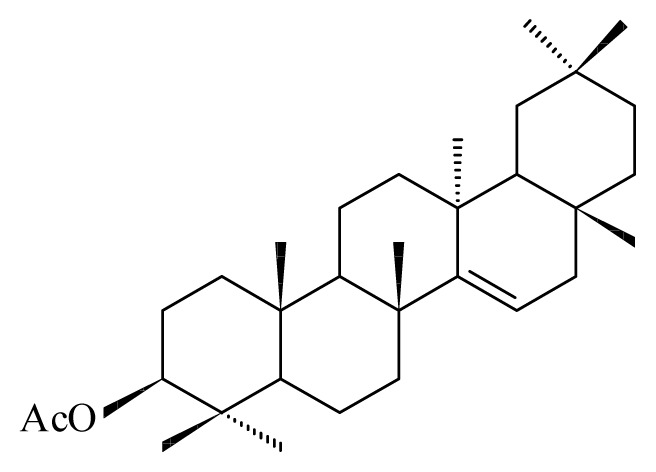 3-O-acetyl aleuritolic acid		

*Homalium africanum*	Salicaceae	Leaves	Hexane Methylene chloride		*O. ochengi*: Microfilaricide	[27]

*Margaritaria discoidea*	Euphorbiaciaea	Roots Leaves	Hexane Methylene chloride		*O. ochengi*: Microfilaricide	

*Anogeissus leiocarpus*	Combretaceae	Bark, leaves	Ethanol	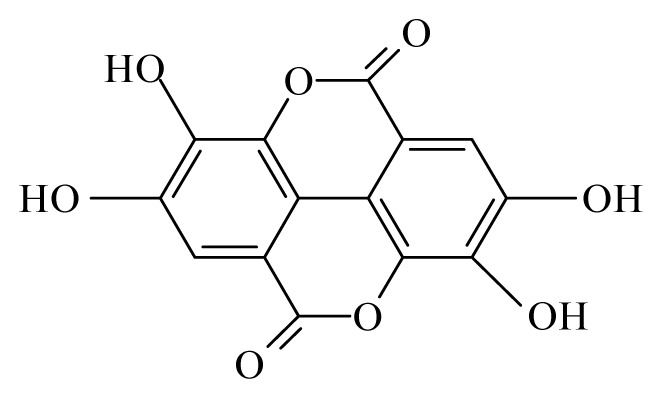 Ellagic acid	*O. ochengi*: Microfilaricide and macrofilaricide. *C. elegans:* High activity on adults and larvae	[28,29]
				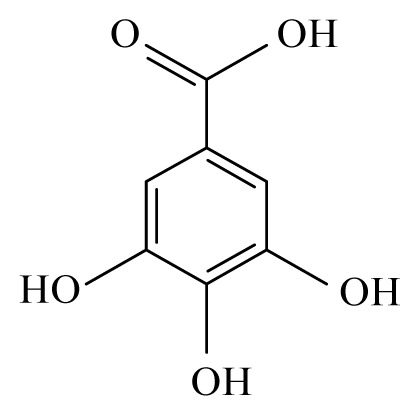 Gallic acid		
				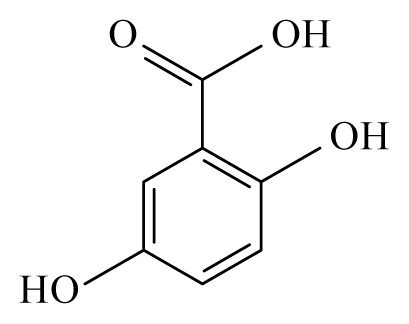 Gentisic acid		

*Khaya senegalensis*	Meliaceae	Bark, leaves	Ethanol		*O. ochengi*: Microfilaricide and macrofilaricide. *C. elegans*: Moderate activity on adults and larvae	[28]

*Euphorbia hirta*	Euphorbiaciaea	Leaves	Ethanol		*C. elegans*: Moderate activity on adults and larvae	

*Parquetina nigrescens*	Asclepiadaceae		Water			[28]

*Annona senegalensis*	Annonaceae		Water		*C. elegans*: Moderate activity on adults and larvae	[28]

*Hagenia abyssinica*	Rosaceae	Female flowers	80% Methanol			[32]

*Acer rubrum*	Aceraceae					
*Rosa multiflora*	Rosaceae					
*Quercus alba*	Fagaceae	Leaves	70% Acetone		*C. elegans*: Moderate activity on adults and larvae	[33]
*Rhus typhina*	Anacardiaceae					

*Lespedeza cuneata*	Fabaceae					
*Salix X sepulcralis*	Salicaceae	Leaves	70% Acetone		*C. elegans*: Low activity on adults and larvae	
*Robinia pseudoacacia*	Fabaceae					
*Botryocladia leptopoda*	Rhodymeniaceae	Whole alga	95% Ethanol		*L. sigmodontis* and *A. viteae*: Adults *B. malayi*: Macrofilaricide and sterilization of female	[34]

*Neurolaena lobata*	Asteraceae	Leaves	Ethanol		*B. pahangi*: Macrofilaricide and micrifilaricide	[35]

*Lantana camara*	Verbenaceae	Stem	95% Ethanol		*A. viteae*: Microfilaricide (95.04%) and sterilization of female (60.66%)	[36]
					*B. malayi: Mastomys coucha* killed 43.05% of the adult and sterilized 76% females	

*Lantana camara*	Verbenaceae	Stem	Chloroform fraction	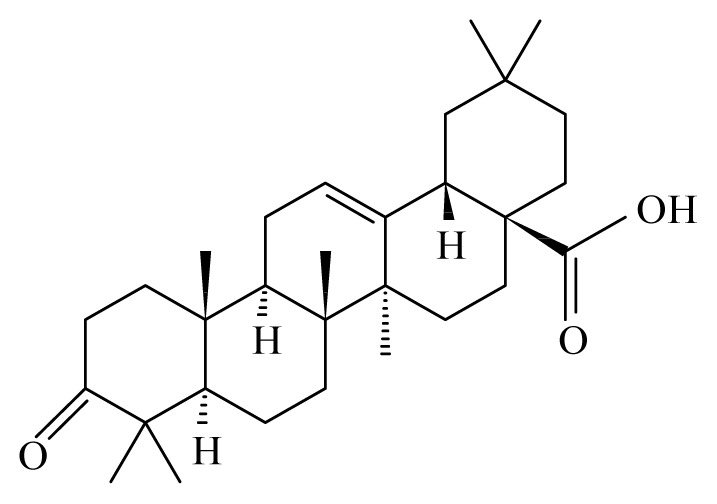 Oleanonic acid	*B. malayi:* in *M. coucha* (*Meriones unguiculatus*) killed 100% (80%) of the adult	[36]
					*B. malayi:* Macrofilaricide	

*Butea monosperma*	Fabaceae	Leaves	Water	Polyphenol	*B. malayi:* Strong inhibition of motility microfilariae, presence of oxidative parameters	[36–38]
		Roots	Water		*B. malayi:* Strong inhibition of motility microfilariae	[36,37]

*Vitex negundo*	Lamiaceae	Roots	Ethanol	Alkaloids, saponin, flavonoids, polyphenol	*B. malayi:* Strong inhibition of motility microfilariae, presence of oxidative parameters	
*Aegle marmelo*	Rutaceae	Leaves	Ethanol	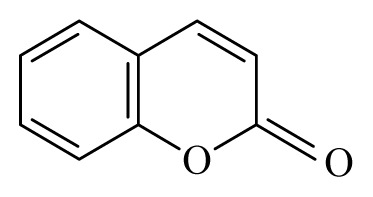 Coumarin Polyphenol	*B. malayi:* Strong inhibition of motility microfilariae, presence of oxidative parameters	[36–38]
*Ricinus communis*	Euphorbiaceae	Leaves	70% Methanol		*B. malayi:* Moderate inhibition of motility microfilariae	

*Caesalpinia bonducella*	Caesalpiniaceae	Seed kernel	Ethanol		*L. sigmodontis* in cotton rats *S. hispidus:* Reduction to up to 96% filariae and 100% female sterilization. Microfilaricide in *B. malayi*.	
			Butanol fraction		*L. sigmodontis* in cotton rats *S. hispidus:* Reduction to up to 73.7% microfilariae. 82.5% mortality of macrofilaria and 100% female sterilization. Microfilaricide in *B. malayi*.	[39]
			Aqueous fraction		*L. sigmodontis* in cotton rats *S. hispidus:* Reduction to up to 90% microfilariae. 82.5% mortality of macrofilaria and 100% female sterilization. Microfilaricide in *B. malayi*.	

*Trachyspermum ammi*	Apiaceae	Fruits	Methanol	Phenolic monoterpene	*S. digitata*: Macrofilaricide *B. malayi:* Macrofilaricide and females sterilization	[40]

*Piper betle*	Piperaceae	Leaves	Methanol	n-Hexane and chloroform fractions	*B. malayi*: Microfilaricide, moderate activity on macrofilariae and female sterilization. Immunomodulatory properties in mices	[41]

*Xylocarpus granatum*	Meliaceae	Fruits	50% Ethanol	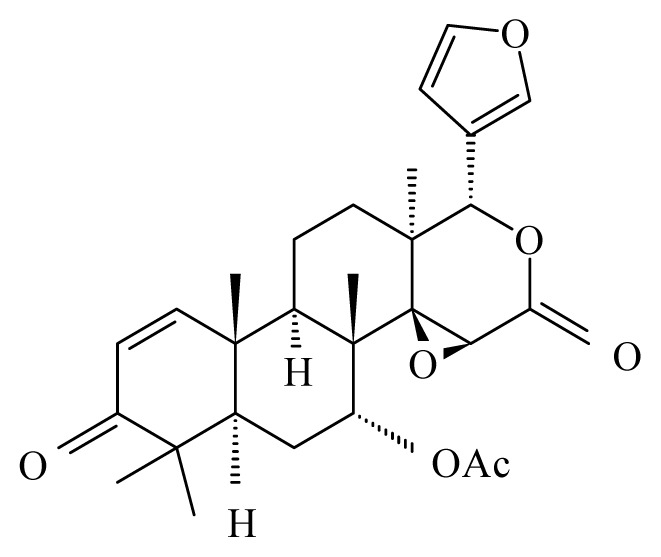 Gedunin	*B. malayi*: Excellent microfilaricidal and macrofilaricidal efficacies	[42,43]
				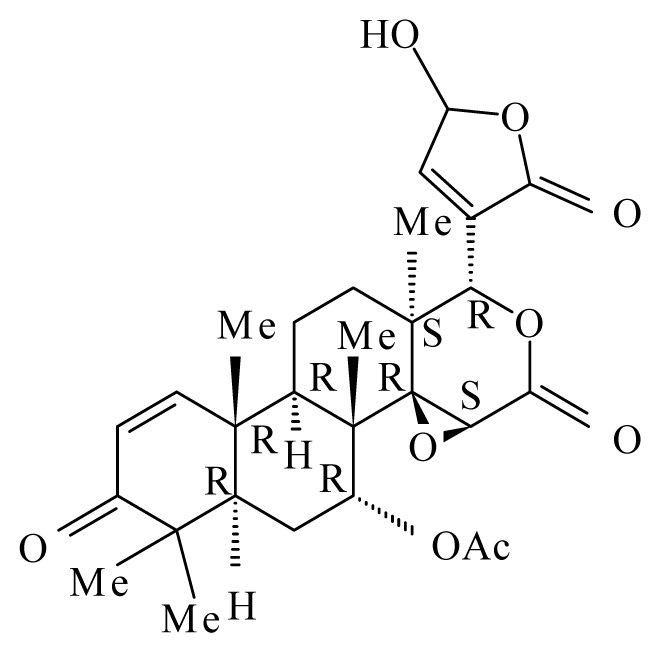 Photogedunin		

*Bauhinia racemosa*	Caesalpinaeceae	Leaves	95% Ethanol	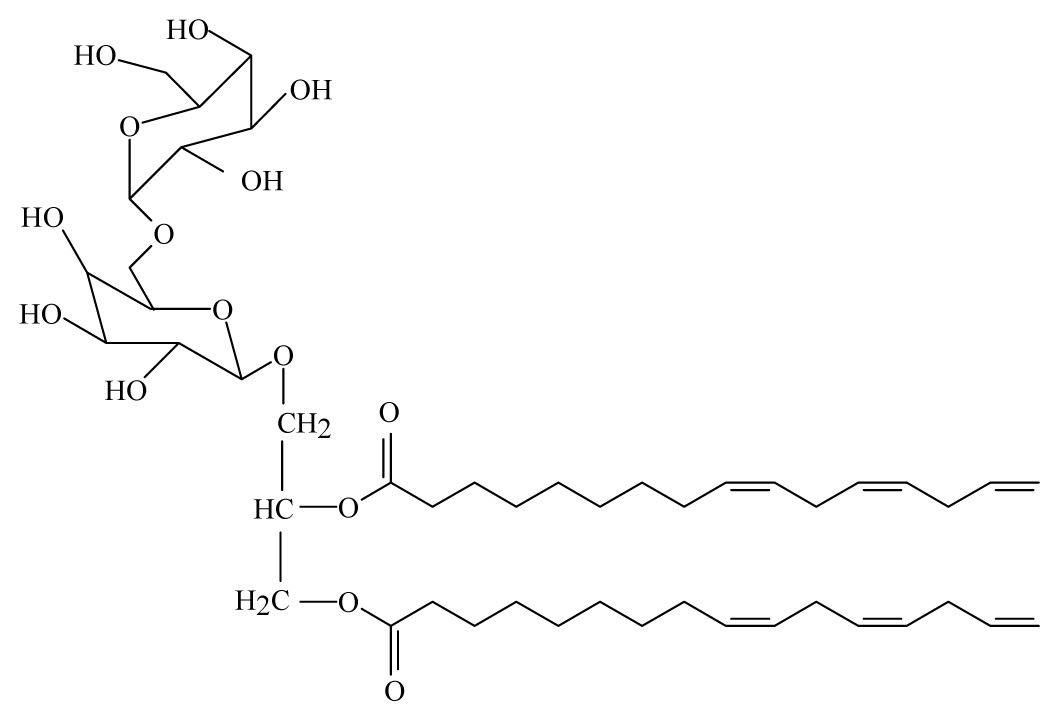 Galactolipid 1	*B. malayi: In vivo* and *in vitro* antifilarial activity	[44]

*Bauhinia racemosa*	Caesalpinaeceae	Leaves	95% Ethanol	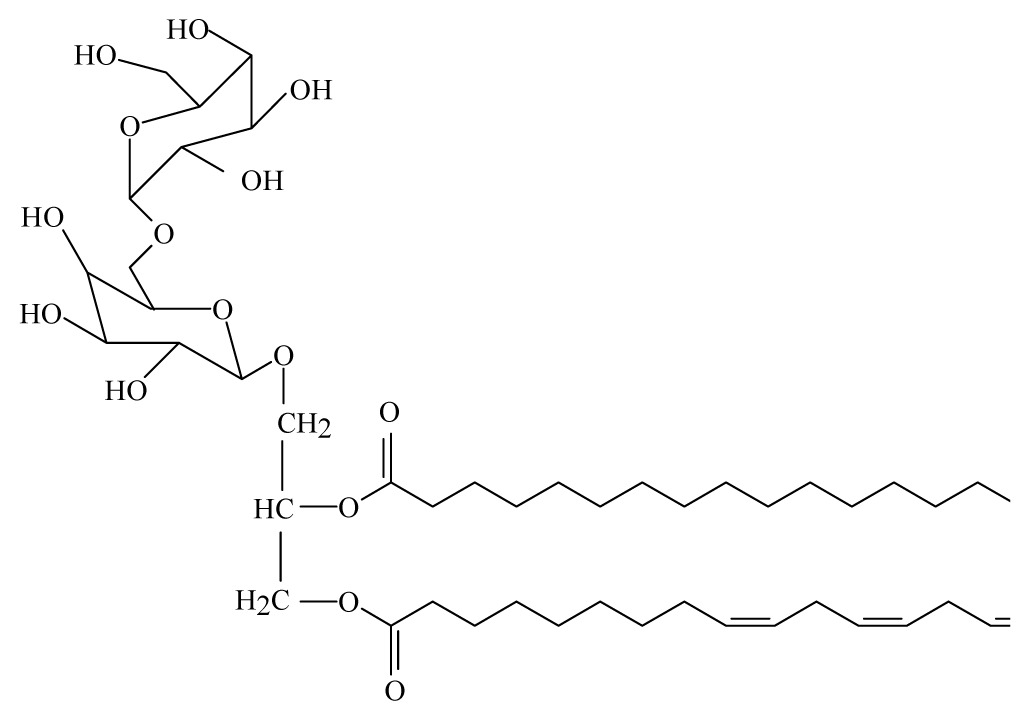 Galactolipid 2	*B. malayi: In vivo* and *in vitro* antifilarial activity	[44]
				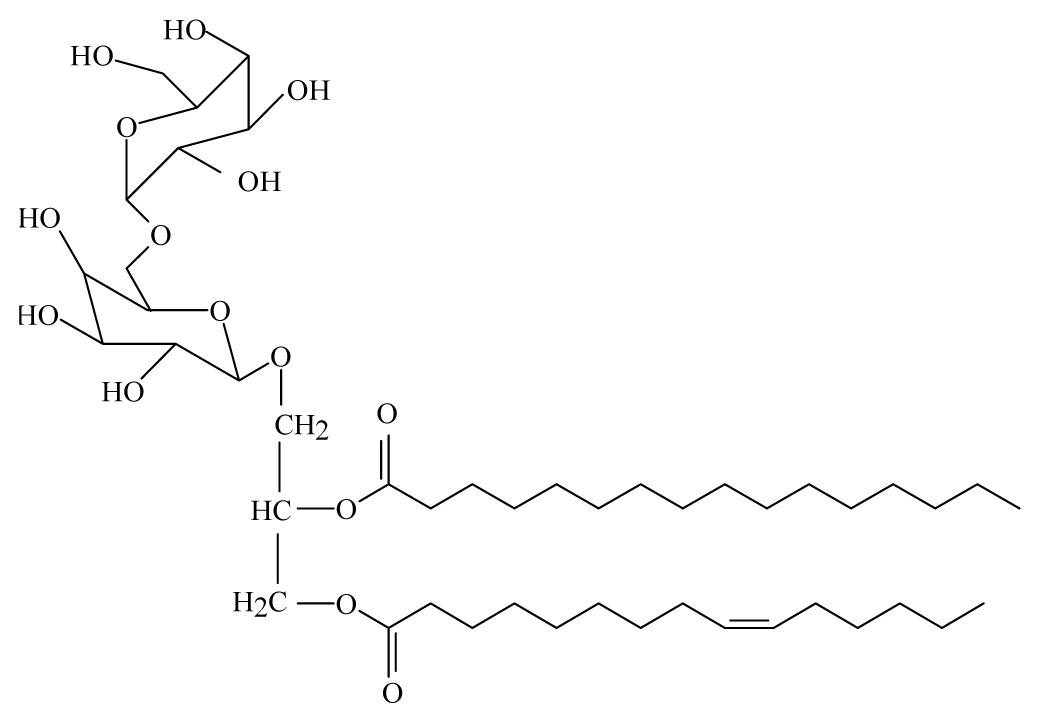 Galactolipid 3		

Corinder: *Coriandrum sativum*	Apiaceae			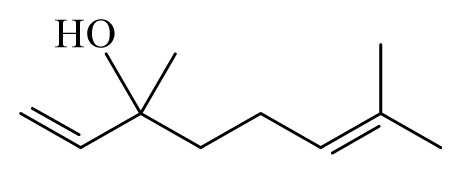 Linalool		[45]
				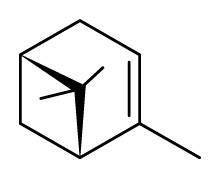 α-Pinene		

Cassia: *Cassia*	Fabaceae			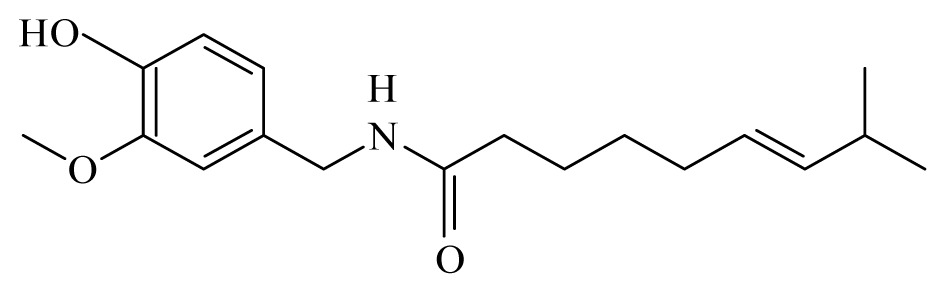 Capsaicin		[45]
Turmeric: *Curcuma longa*	Zingiberaceae			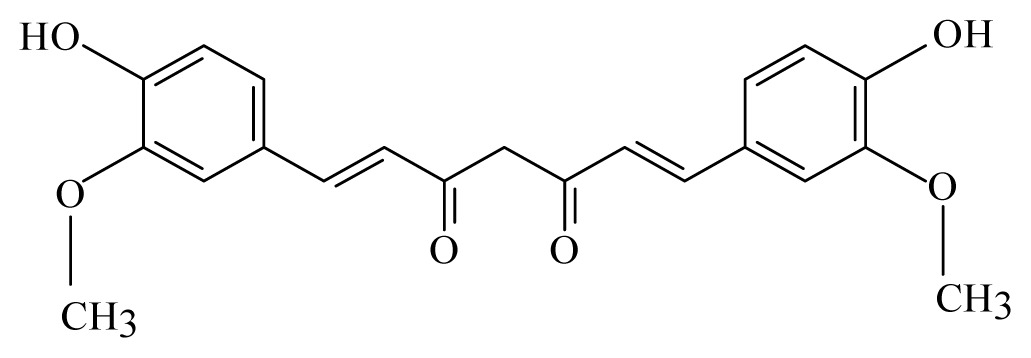 Curcumin		
Allspice: *Pimenta dioica*	Myrtaceae			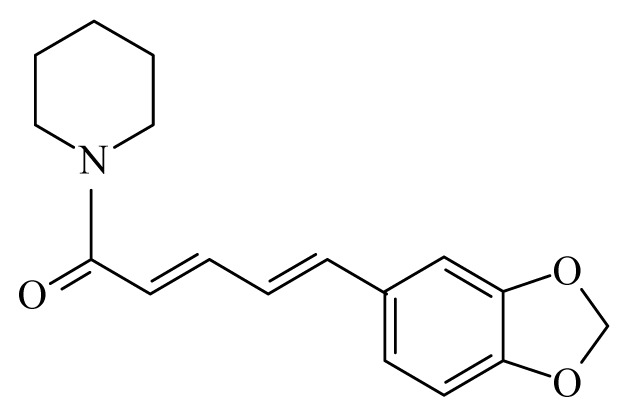 Piperine		
				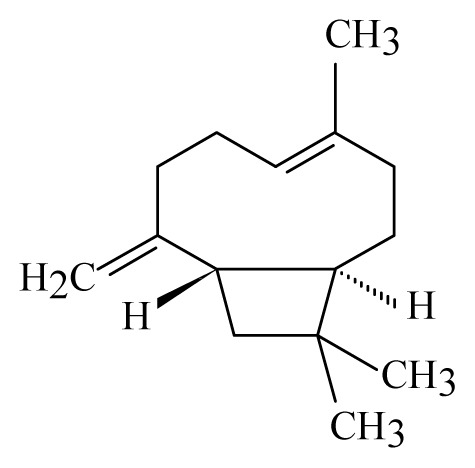 β-Caryophyllene		

Cinnamon: *Cinnamomum*	Lauraceae			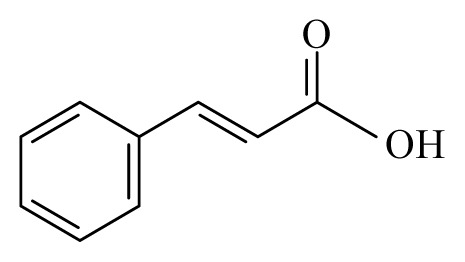 Cinnamic acid β-Caryophyllene	*B. malayi*: GST inhibitory activity *in vitro*	[45]
Strychnous: *Strychnos*	Loganiaceae			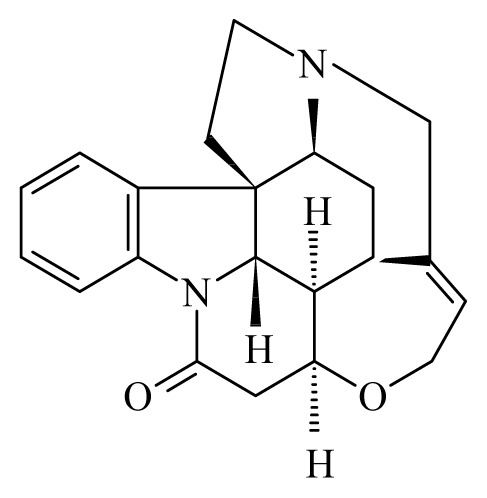 Strychnine		
Lemongrass: *Cymbopogon*	Poaceae			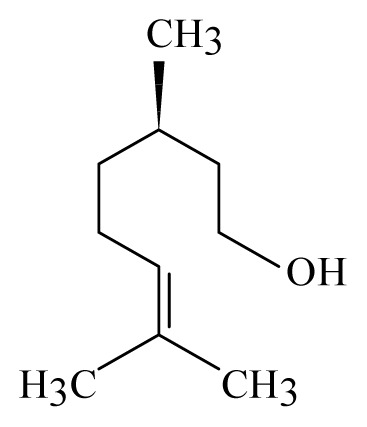 Citronellol		
				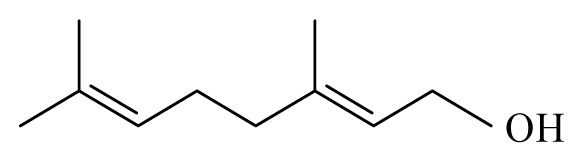 Geraniol		
Garlic: *Allium sativum*	Amaryllidaceae			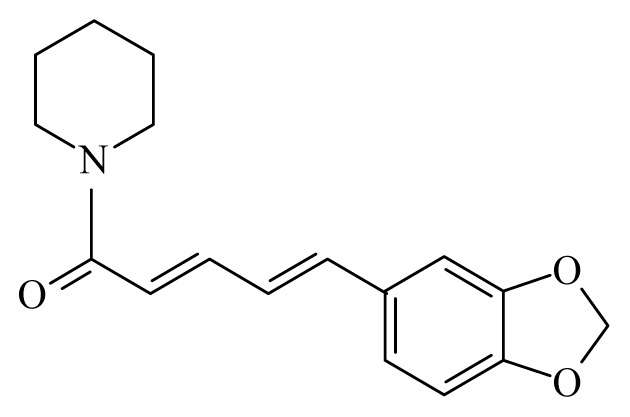 Piperine		

Litsea: *Litsea*	Lauraceae			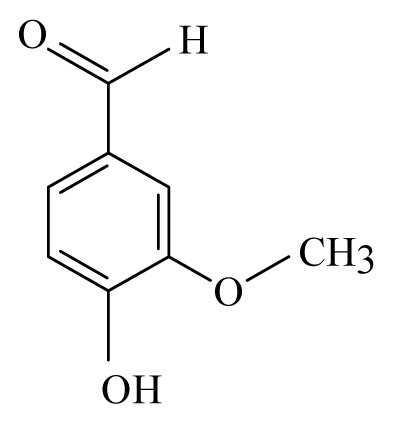 Vanillin		[45]
Vanilla: *Vanilla*	Orchidaceae			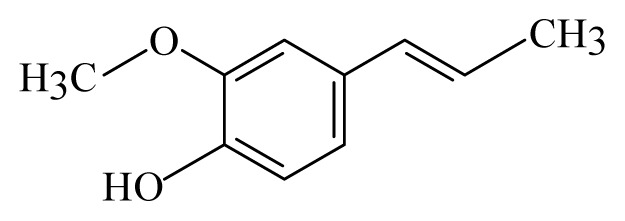 Isoeugenol		

*Withania somnifera*	Solanaceae Roots			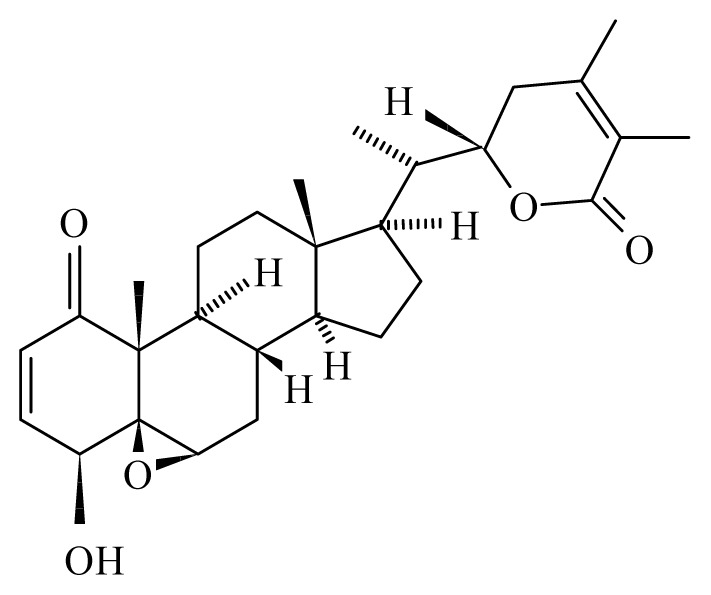 Withanolide	Protection to the rodent host *M. coucha* against infection of filarial parasite *B. malayi*	[46,47]

*Nigella sativa*	Ranunculaceae	Seeds			*B. malayi*: Immunomodulatory and therapeutic properties in mices	[48]

Galactolipid 1: (2*S*)-1, 2-di-*O*-linolenoyl-3-*O*-*a*-galactopyra-nosyl-(1/6)-*O*-*b*-galactopy-ranosyl glycerol; Galactolipid 2: (2*S*)-1-O-linolenoyl-2-*O*-palmitoyl-3-*O*-*a*-galacto-pyranosyl-(1/6)- *O*-*b*-galactopyranosyl glycerol; Galactolipid 3: (2*S*)-1-*O* oleoyl-2-*O*-palmitoyl-3-*O*-*a*-galacto-pyranosyl-(1/6)-*O*-*b*-galactopyranosyl glycerol.

**Table 2:** List of medicinal plants, products and active compounds known to have anti-schistosomal properties.

Compounds/Substances	Origin	Schistosomicidal activities			Assays		References
		*in vitro*	*in vivo*	Observations	Toxicity	Clinical	
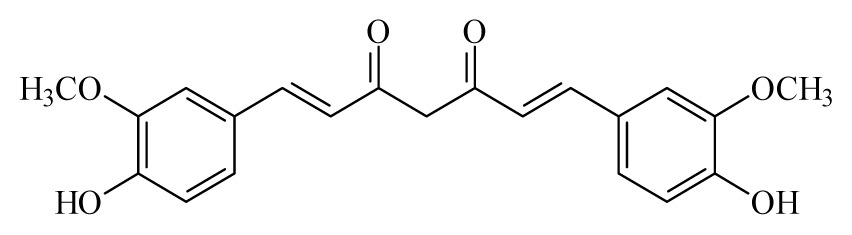 Curcumin	*Curcuma longa*	*S. mansoni* adult—50 μM (100% mortality in male and female)	400mg/Kg in mouse 1—43.5% mortality in male and 4.6% in female of *S. mansoni*)	Reduction in the oviposition Induced separation of males and females Reduction in the motor activity	nd	nd	[99,113]
Essencial oil (sesquiterpenes 57.20% and monoterpenes 42.13%)	*Plectranthus neochilus*	*S. mansoni* adult—LC_50_-value 89.65 mg/mL at 24 h LC_50_-value 58.18 mg/mL at 120 h	nd	Reduction in the oviposition Induced separation of males and females Reduction in the motor activity	Non-toxic in V79 cells^2^ (concentrations lower than 200 μg/mL)	nd	[101]
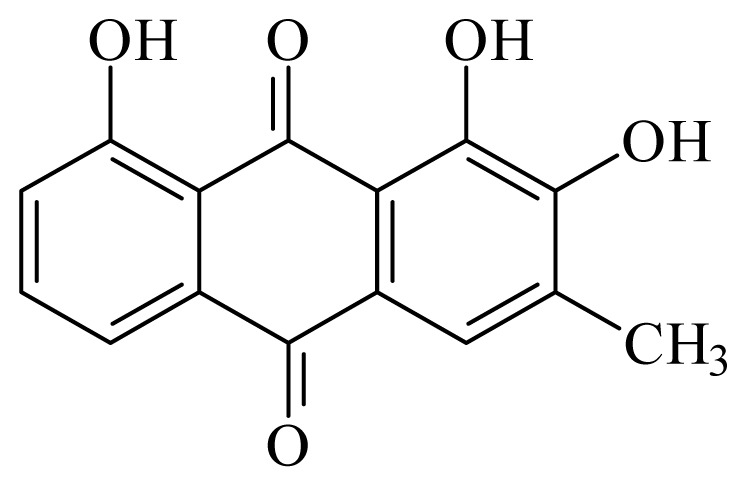 2-hydroxychrysophanol	*Hemerocallis fulva*	*S. mansoni* adult—50 μg/mL (35% mortality in male and female) Schistosomula—25 μg/mL (80% mortality)	nd	nd	nd	nd	[114]
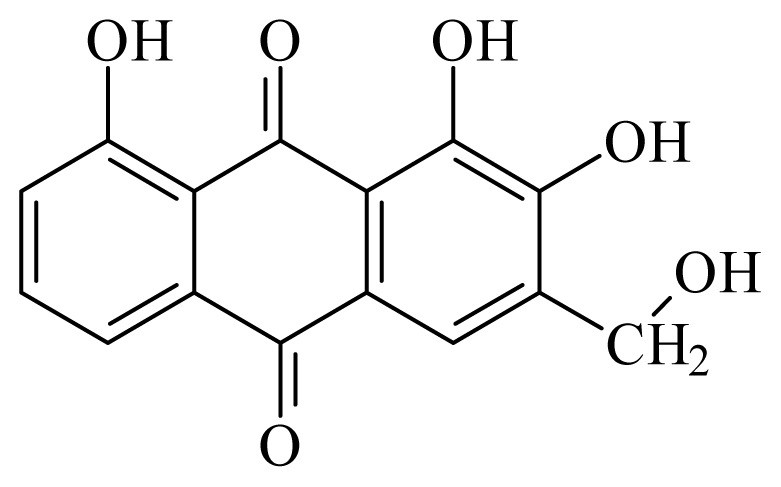 Kwanzoquinone E	*Hemerocallis fulva*	*S. mansoni* adult—50 μg/mL (55% mortality in male and female) Schistosomula—25 μg/mL (100% mortality)	nd	nd	nd	nd	[114]
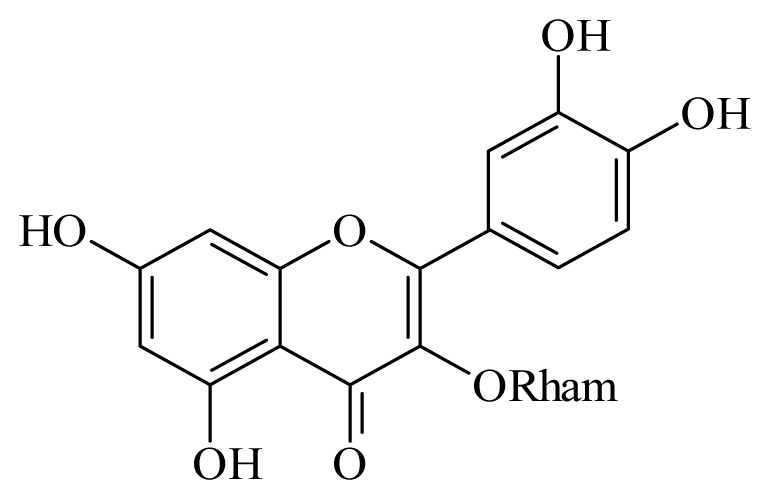 Quercetin 3-*O*-β-D-rhamnoside	*Schefflera vinosa*	*S. mansoni* adult—100 μM (25% mortality in male and female)	nd	Reduction in the Motor activity	nd	nd	[98]
Methanol leaves extract	*Cleome droserifolia*	Nd	Doses of 0.31 g kg^−1^ for 21 days*—*32.46% reduction of *S. mansoni* in mice 3	nd	nd	nd	[115]
Aqueous leaves extract	*Clerodendrum umbellatum*	Nd	80 mg/kg in mice 4—100% mortality in *S. mansoni*	Reduction in the oviposition (75.49% released eggs in faeces)	nd	nd	[116]
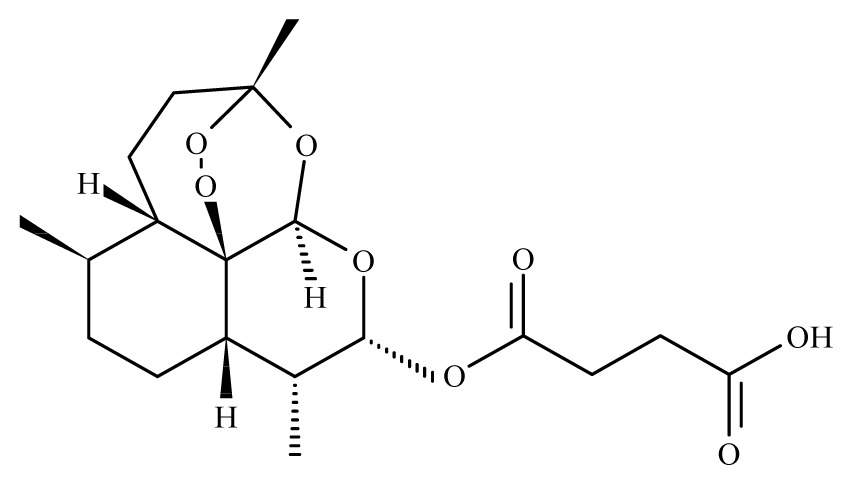 Artesunate	*Artemisia annua*	*S. mekongi* adult—40 μg/mL (100% mortality in male and female) *S. mansoni* adult—40 μg/mL (80% mortality in male and female)	150 to 300 mg/kg in mice—67 and 77% mortality in male and female)	Reduction in the motor activity Reduction in the oviposition Tegumental disruption	nd	nd	[117,118]
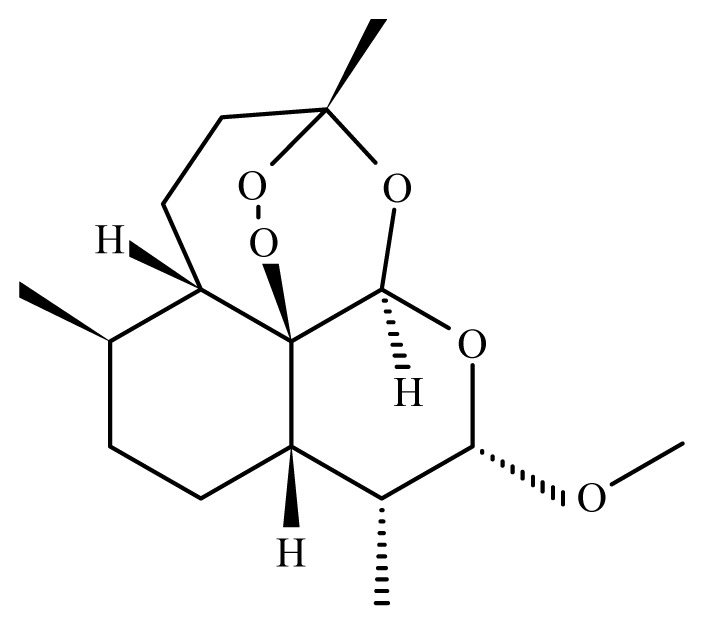 Artemether	*Artemisia annua*	Nd	50 mg/Kg in mice 5—reduction of *S. mansoni* female 100 mg/Kg in mice—61.5% mortality in *S. mansoni* female 150 to 300 mg/kg in mice—88 and 97% mortality in *S. mansoni* male and female 10 mg/Kg in rabbit 6*—*97% mortality in *S. japonicum* male and female 10 mg/Kg in dog 6—99.3% mortality in *S. japonicum* male and female	Reduced liver and spleen weight of treated animals Reduction in the motor activity Tegumental disruption Alteration of the reproductive organs, ovarian volume reduction and depigmentation of the intestinal parasites portion	nd	30 mg/kg (2 oral doses)	[109,110, 118–120]
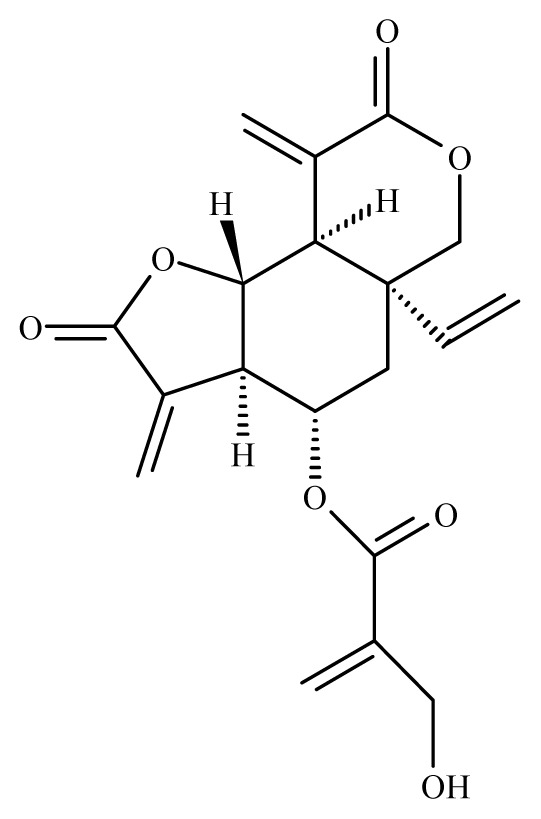 Vernodalin	*Vernonia amygdalina*	*S. japonicum* adult —20 μg/mL (100% immobilization and oviposition)	2.5 mg/kg in mice – no mortality *S. japonicum*	Inhibition of the oviposition Inhibition of the motor activity	Toxic in KB, P-388 L-1210 cells^7^ at 120 mg/Kg	nd	[104]
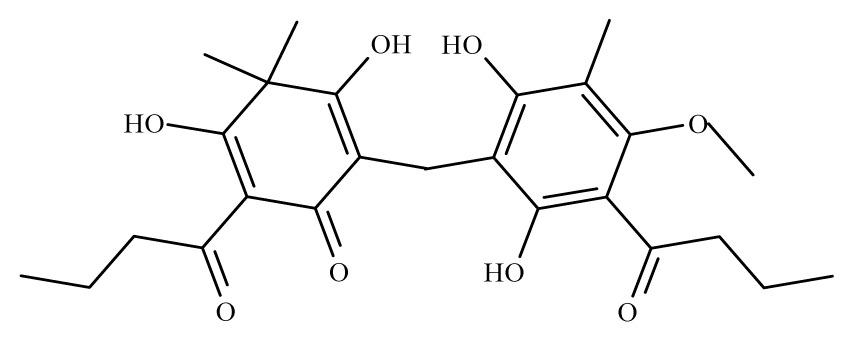 Aspidin	*Dryopteris spp.*	*S. mansoni* adult—25 μM (100% mortality in male and female)	nd	Reduction in the motor activity Tegumental alterations	nd	nd	[100]
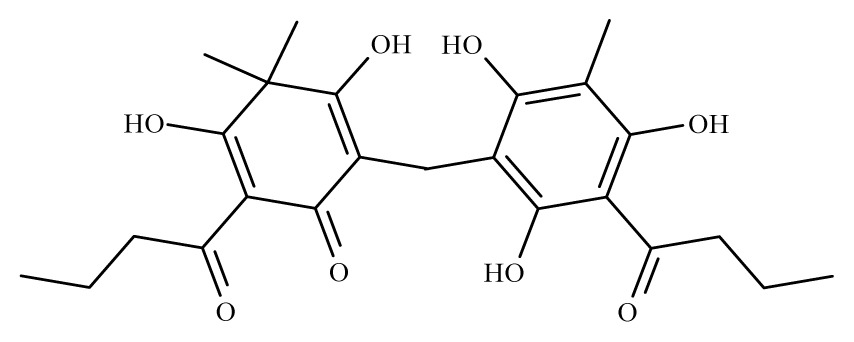 Favaspidic acid	Dryopteris *spp.*	*S. mansoni* adult—50 μM (100% mortality in male and female)	nd	Reduction in motor the activity Tegumental alterations	nd	nd	[100]
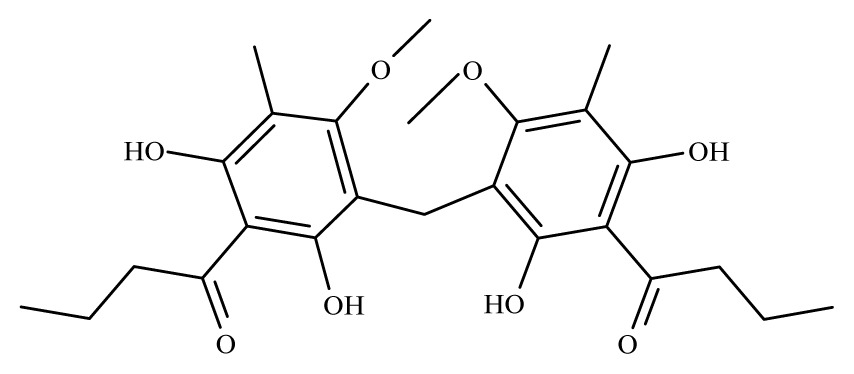 Methylene-bis-aspidinol	Dryopteris *spp*.	*S. mansoni* adult—100 μM (100% mortality in male and female)	nd	Reduction in the motor activity	nd	nd	[100]
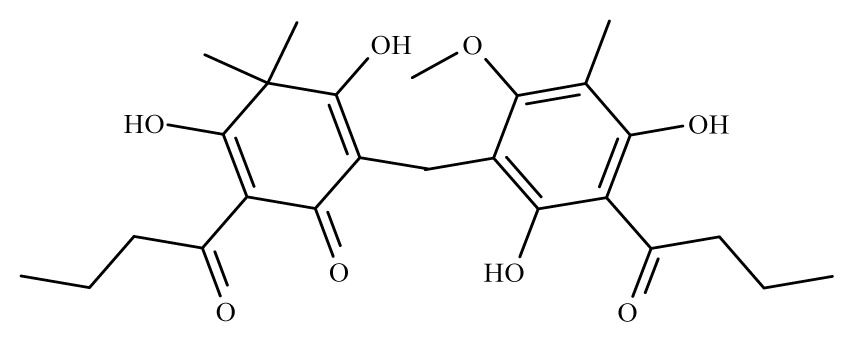 Desaspidin	*Dryopteris spp.*	*S. mansoni* adult—25 μM (100% mortality in male and female)	nd	Reduction in the motor activity	nd	nd	[100]
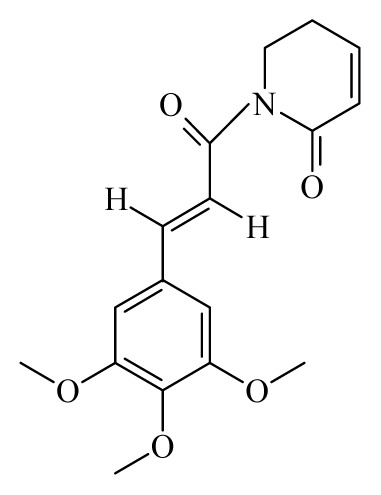 Piplartine	*Piper tuberculatum*	*S. mansoni* adult—15.8 μM (100% mortality in male and female) Schistosomula—5 μM (100% mortality)	nd	Reduction in the motor activity Reduction in the oviposition	Non-toxic in Vero cell 8 at 31.5 μM	nd	[92,103]
Methanol leaves extract	*Baccharis trimera*	*S. mansoni*—130 μg/mL (100% mortality in male, female and schistosomula	nd	Reduction in the motor activity Tegumental alterations	Non-toxic in human keratinocytes cell line at 250 μg/mL	nd	[121]
Avocado/soybean unsaponifiables	*Persea americana*	Nd	300 mg/kg—3 oral doses in mice—30% *S. mansoni* mortality	Reduction in the motor activity Tegumental alterations Reduction in the oviposition	nd	nd	[122]
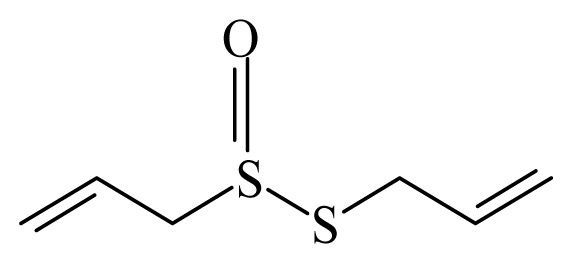 Allicin	*Allium sativum*	*S. mansoni* adult—20 mg/mL—no mortality	nd	5mg/mL tegumental alterations 10mg/mL changes in tubercles, loss or changes in the spines; 15 and 20mg/mL tegumental disruption (vesicle and ulceration)	nd	nd	[123]

1 Male CD1 albino—16 injections and total dose of 400 mg/kg;

2 Chinese hamster lung fibroblasts;

3 Swiss albino;

4 Male Balb/c—daily doses for 14 days;

5 Female Balb/c—doses administered at day 0, 20 and 60 post infection; Rats - intramuscular dose;

6 Rabbits and dogs treated 7, 14, 21, 28, 35 days post infection;

7 KB-Cell line of human oral carcinoma, P388-murine leukemia, L1210-Mouse lymphocytic leukemia cells;

8 African green monkey kidney fibroblast.

**Table 3:** List of medicinal plants, products and active compounds known to have anti-trypanosomal properties.

Compounds/Substances	Origin	Trypanocidal activities			Assays		References
		*in vitro*	*in vivo*	Observations	Toxicity	Clinical	
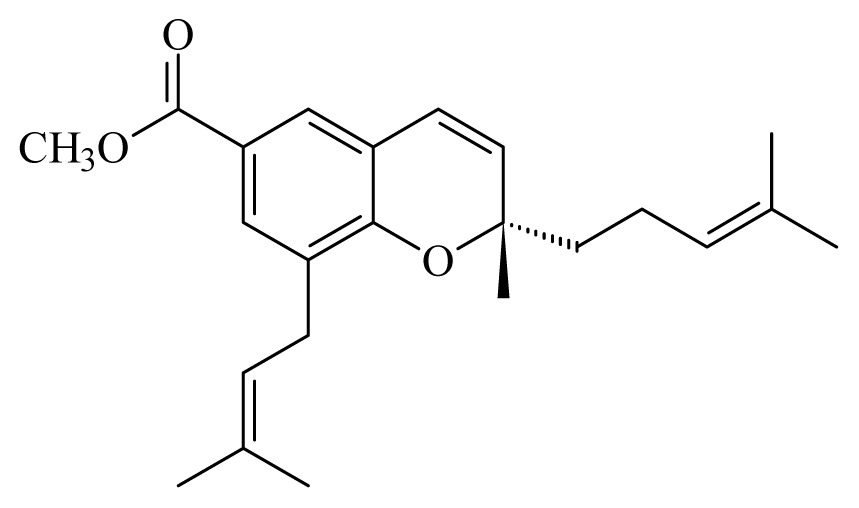 [(2*S*)-methyl-2-methyl-8-(3-methylbut-2′-enyl)-2-(4-methylpent-3-enyl)-2*H*chromene-6-carboxylate]	*Piper*^1^	*T. cruzi* epimastigote—IC_50_-value 2.82 μM	nd	nd	nd	nd	[143]
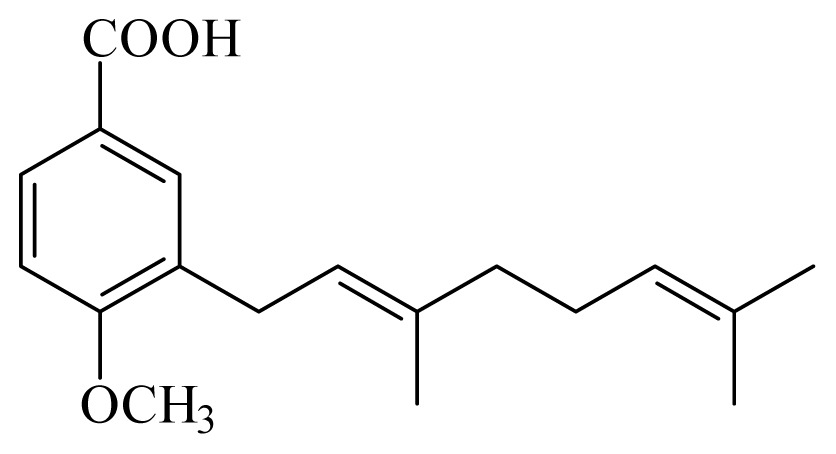 3-(3,7-dimethyl-2,6-octadienyl)-4-methoxy-benzoic acid	*Piper aduncum*	*L. braziliensis* promastigote—IC_50_-value 6.5 μg/mL	nd	nd	nd	nd	[144]
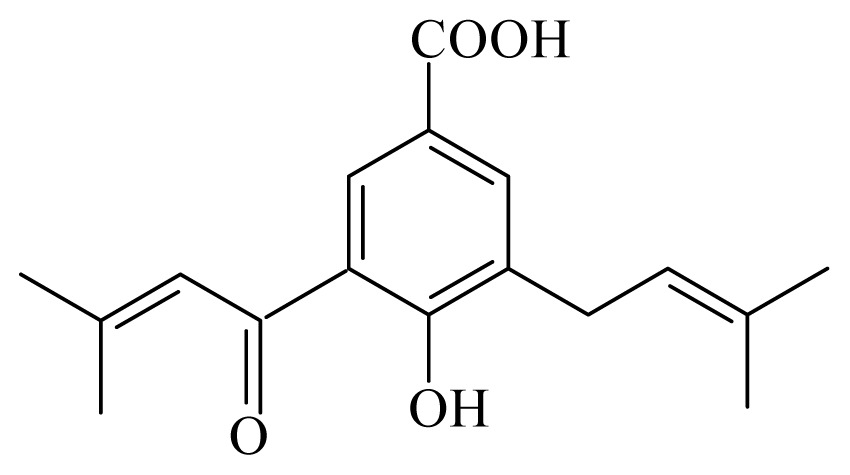 4-hydroxy-3-(3-methyl-1-oxo-2-butenyl)-5-(3-methyl-2-butenyl)benzoic acid	*Piper aduncum*	*T. cruzi* epimastigote—IC_50_ 16.5 μg/mL	nd	nd	nd	nd	[144]
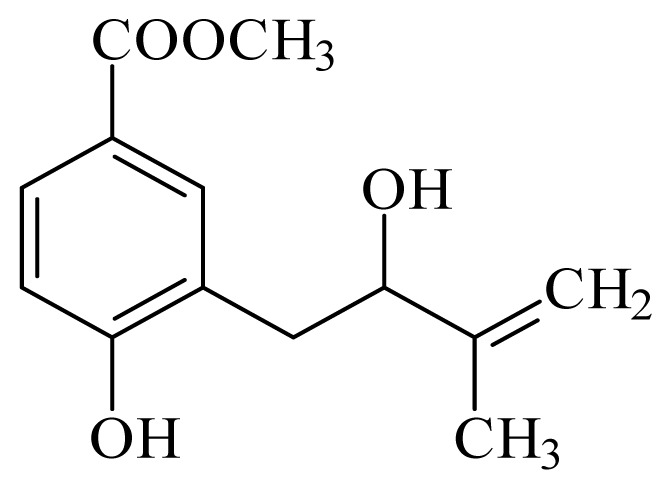 methyl 4-hydroxy-3-(2-hydroxy-3-methyl-3-butenyl)benzoate	*Piper glabratum*	*T. cruzi* epimastigote—IC_50_-value 15.6 μg/mL	nd	nd	nd	nd	[145]
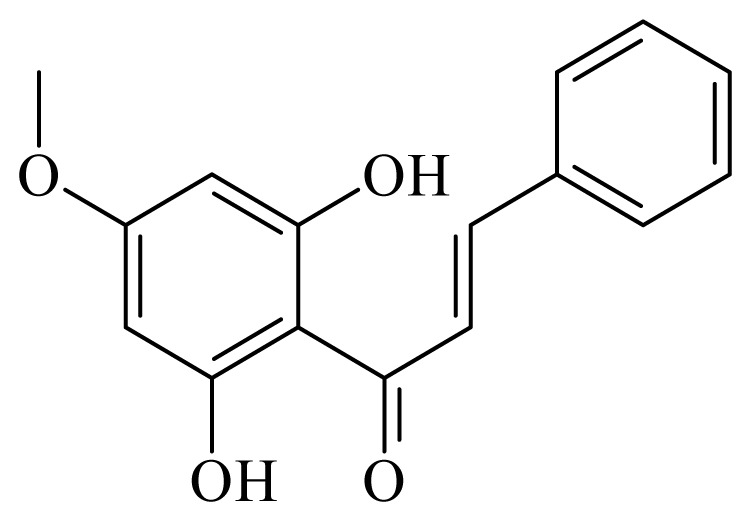 2,6-dihydroxi-4-metoxichalcone	*Piper aduncum*	*L. amazonensis* promastigote—IC_50_-value 0.5 μg/mL amastigotes—IC_50_-value 24 μg/mL	nd	nd	nd	nd	[146]
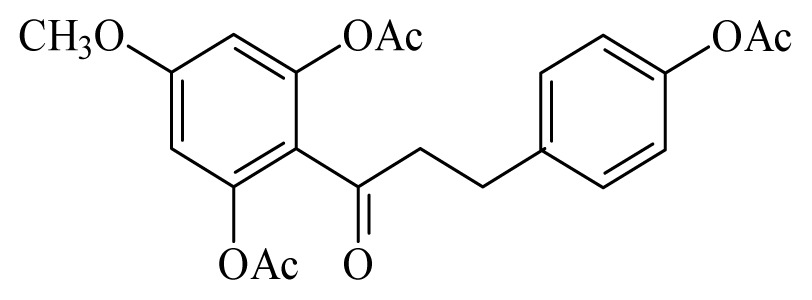 dihydrochalcone	*Piper elongatum*	*L. braziliensis* promastigote—IC_50_-value 2.98 μg/mL	nd	nd	nd	nd	[147]
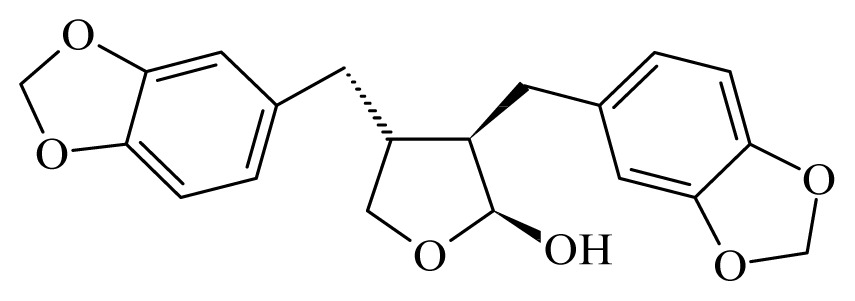 Cubebin	*Piper cueba*	*L. donovani* promastigotes—IC_50_-value 28 μg/mL	100.0 mg/kg, i.p. in hamster infected with *L. donovani*	nd	nd	nd	[148]
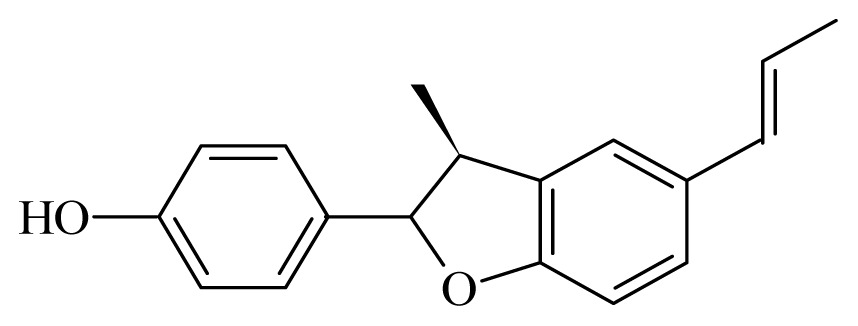 Conocarpan	*Piper regnellii*	*T. cruzi* epimastigote—IC_50_-value 8.0 μg/mL	nd	nd	nd	nd	[149]
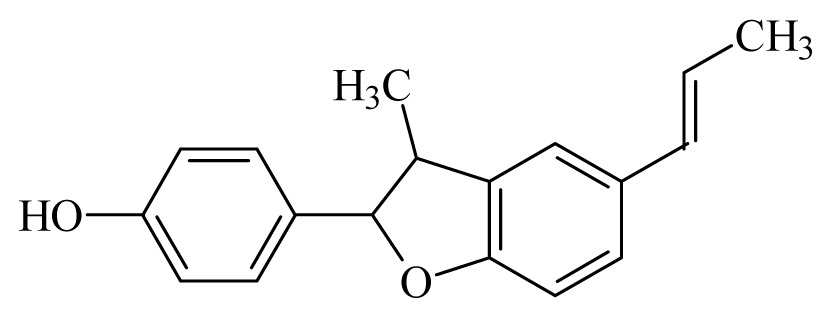 Eupomatenoid	*Piper regnellii*	*T. cruzi* epimastigote—IC_50_-value 7.0 μg/mL	nd	nd	Non-toxic in Vero cells (CC_50_ 250 μg/mL)	nd	[149]
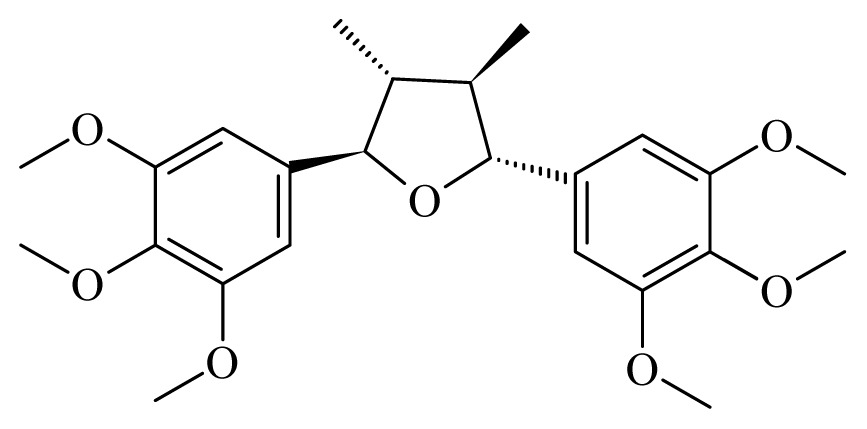 Grandisin	*Piper solmsianum*	*T. cruzi* trypomastigote—IC_50_-value 8.74 μg/mL	nd	nd	nd	nd	[150]
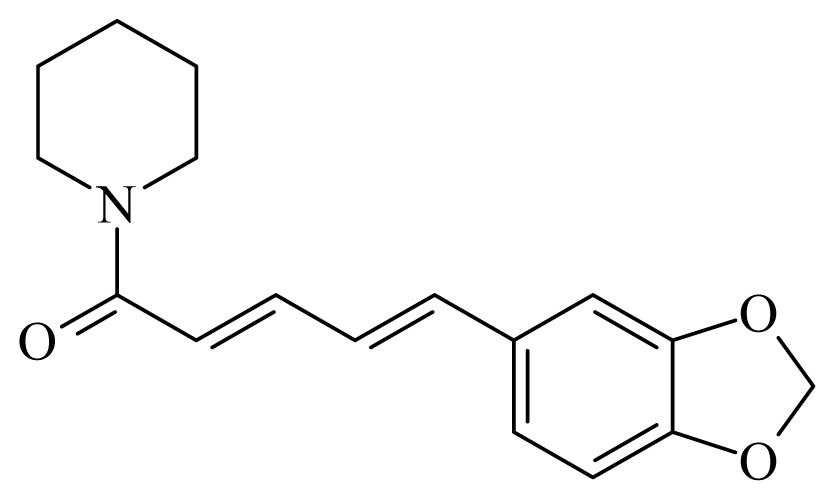 Piperine	*Piper*	*T. cruzi* epimastigote—IC_50_-value 7.36 μM amastigote—IC_50_-value 4.91 μM	nd	nd	nd	nd	[151]
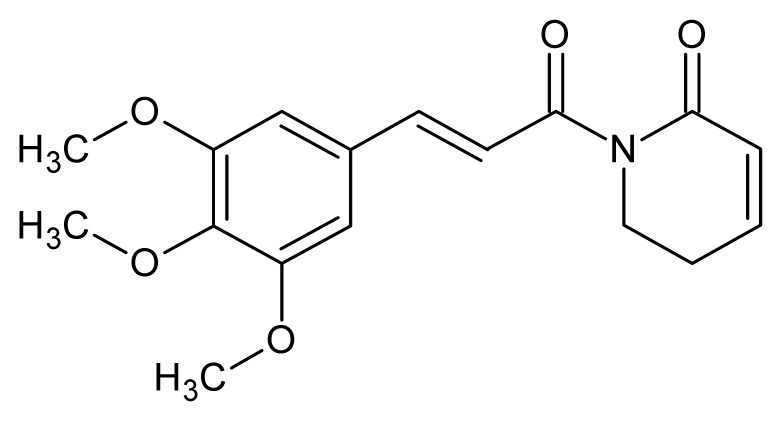 Piplartine	*Piper tuberculatum Piper retrofractum*	*T. cruzi* epimastigote—IC_50_-value 10.5 μM *L. donovani* promastigotes—IC_50_-value 7.5 μg/mL	30 mg/kg ip. in hamster infected with *L. donovani*	nd	nd	nd	[148,152]
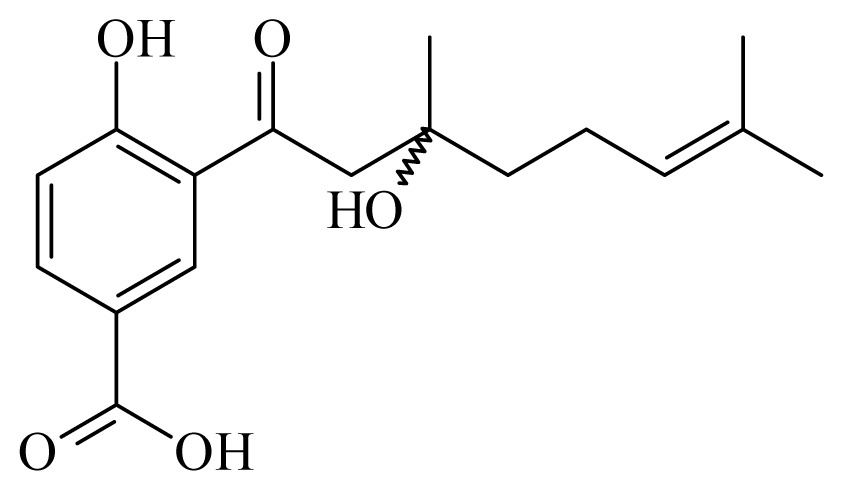 prenylated hydroquinone [1,4-dihydroxy-2-(3′,7′-dimethyl-1′-oxo-2′-E,6′-octadienyl)benzene	*Piper crassinervium*	*T. cruzi* epimastigote—IC_50_-value 6.10 μg/mL	nd	nd	nd	nd	[153]
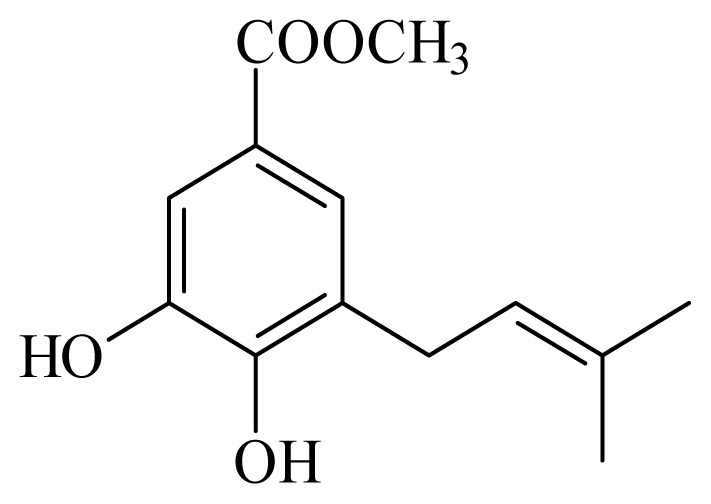 methyl 3,4-dihydroxy-5-(3′-methyl-2′-butenyl)benzoate	*Piper glabratum*	*L. braziliensis*, *L. amazonensis* and *L. dono*v*ani*—IC_50_-value 13.8–18.5 μg/mL)	nd	nd	nd	nd	[145]
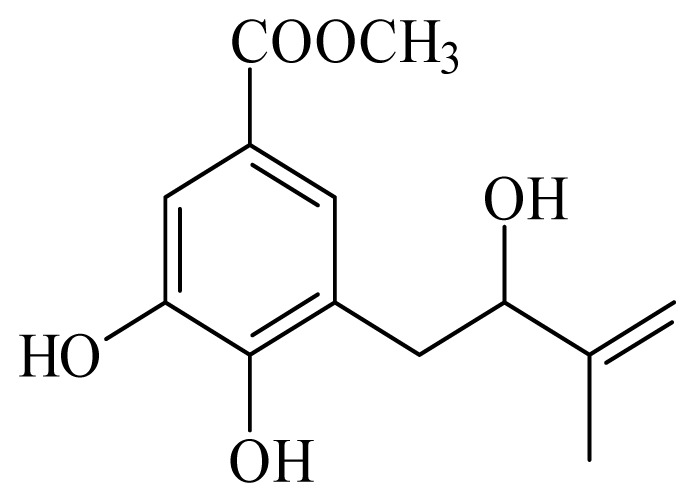 methyl 3,4-dihydroxy-5-(2-hydroxy-3-methylbutenyl)benzoate	*Piper glabratum*	*T cruzi* epimestigote—IC_50_-value 16.4 μg/mL	nd	nd	nd	nd	[145]
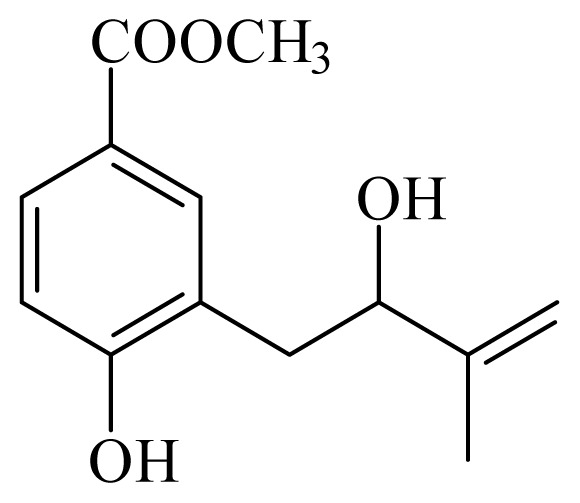 methyl 4-hydroxy-3-(2-hydroxy-3-methyl-3-butenyl)benzoate	*Piper glabratum*	*T cruzi* epimestigote—IC_50_-value 15.6 μg/mL	nd	nd	nd	nd	[145]
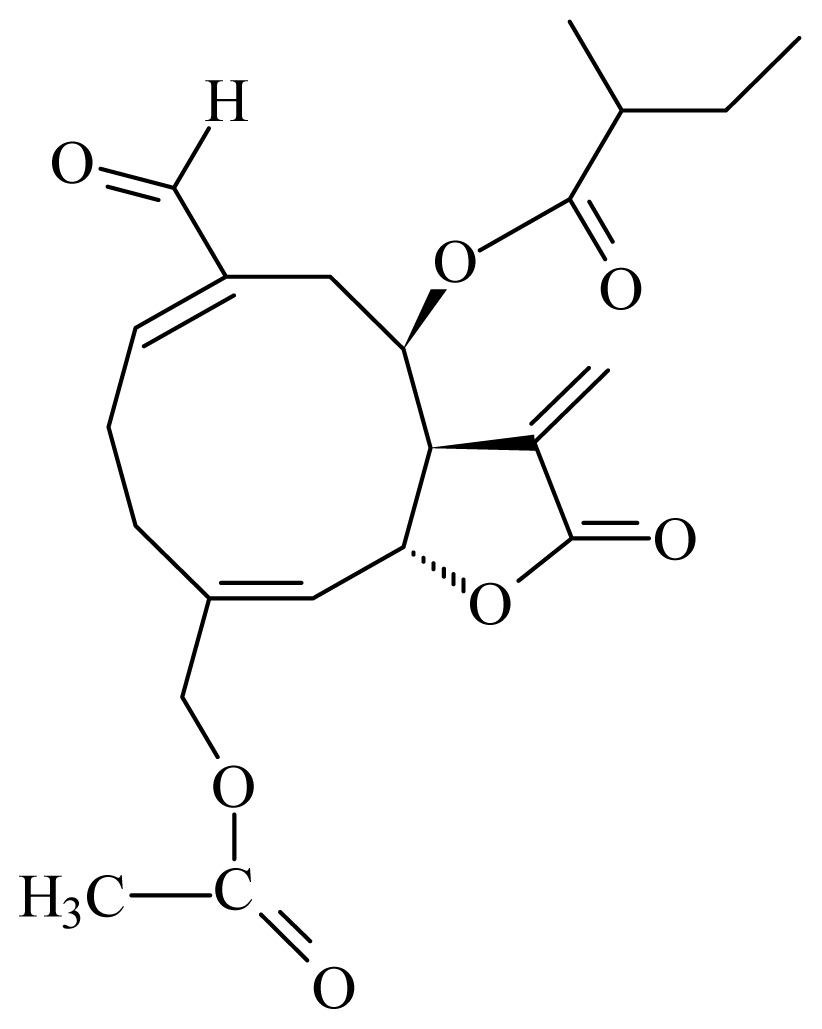 (15-acetoxy-8_-[(2-methylbutyryloxy)]-14-oxo-4,5-cis-acanthospermolide)	*Acanthospermum hispidum*	*T. brucei brucei*—IC_50_-value 2.45 μM *L. mexicana Mexicana* —IC_50_-value 0.94 μM	nd	nd	nd	nd	[154]
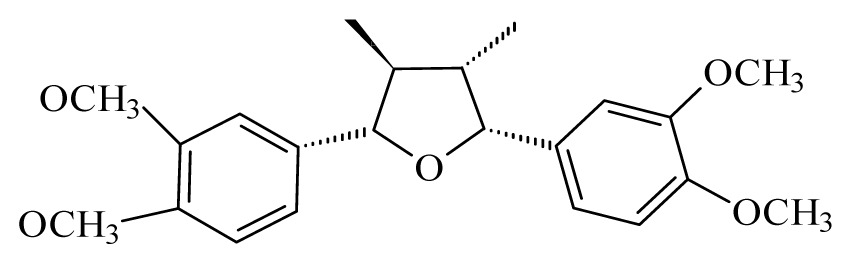 Veraguensin	*Nectandra megapotamica*	*L.donovani* promastigotes—IC_50_-value 18 μg/mL and IC_90_-value 36 μg/mL	nd	nd	Non-toxic in Vero cells^5^ in 10 μg/ml	nd	[91]
Methanol extract from stem bark	*Acacia nilotica*	na	200 mg/kg body weight in mice infected with *T. brucei brucei*	Clear the parasites from circulation within 6 days of treatment	na	na	[136]
Methanol stem bark extract	*Bombax buonopozense*	nd	300 mg/kg body weight in mice infected with *T. brucei brucei*	Clear the parasites from circulation within 7 days of treatment	nd	nd	[136]
Dichloromethane bark extract	*Warburgia salutaris*	*T. brucei brucei*—IC_50_ -value 10.68 μg/mL	nd	nd	nd	nd	[155]
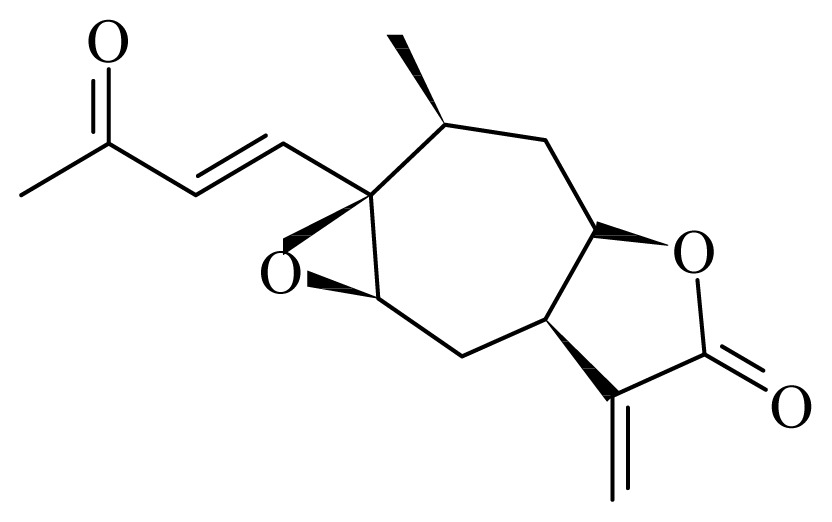 8-epixanthatin 1β,5β-epoxide	*Xanthium brasilicum Vell*	*T. brucei*—IC_50_ 0.09 μg/mL *T. cruzi*—IC_50_-value 2.95 μg/L *L. donovani*—IC_50_ 0.16 μg/mL	nd	nd	nd	nd	[142]

1 Synthetic compound derived from chromenes isolated from *Piper aduncum* and *Piper gaudichaudianum*;

2 Intraperitoneally, twice daily for 3 days;

3 per os, twice daily for 3 days;

4 Balb/c infected with *L. amazonensis* and treated for 28 days;

5 African green monkey kidney fibroblast.
